# Silylative aromatization of *p*-quinone methides under metal and solvent free conditions†

**DOI:** 10.1039/d1ra03193g

**Published:** 2021-05-18

**Authors:** Tingting Li, Yuzhu Wu, Wenzeng Duan, Yudao Ma

**Affiliations:** Department of Chemistry, Shandong University Shanda South Road No. 27 Jinan 250100 P. R. China ydma@sdu.edu.cn; School of Chemistry and Chemical Engineering, Liaocheng University Liaocheng 252000 P. R. China duanwenzeng@lcu.edu.cn

## Abstract

A base-mediated silylation reaction leading to benzyl silanes has been developed. Under transition-metal and solvent free conditions, the silylation of a wide array of *p*-quinone methides is achieved using a Cs_2_CO_3_ catalyst in yields up to 96%. Carboxylation of the as-obtained organosilane with gaseous CO_2_ provides a new synthetic protocol for the preparation of carboxylic acid.

## Abstract 2

A novel and efficient synthetic protocol is reported for the synthesis of benzyl silanes from readily available silylborane and *p*-quinone methides using 5% cesium carbonate under solvent-free conditions.

## Graphical abstract

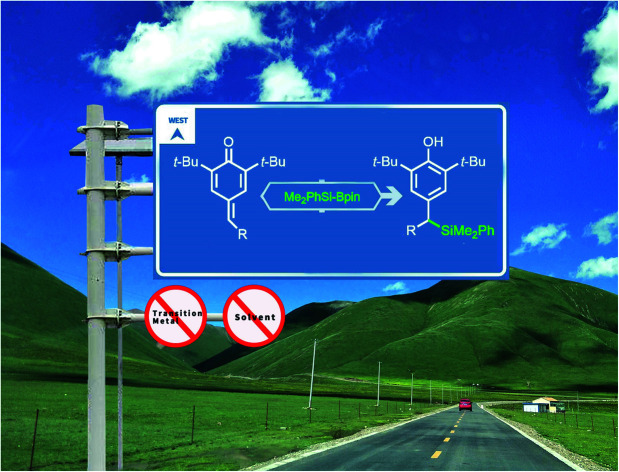

## Introduction

**Scheme 1:**
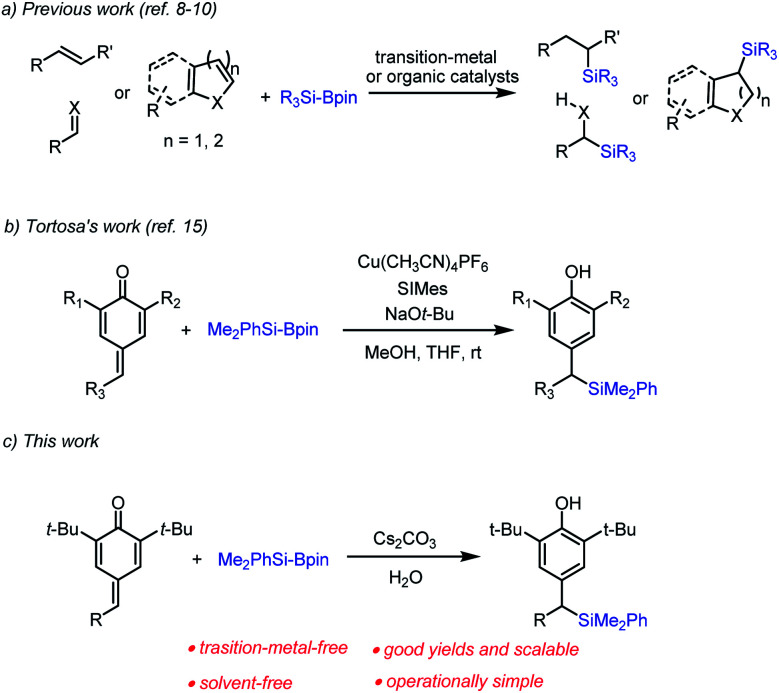
Catalytic nucleophilic addition to unsaturated acceptors.

Organosilicon compounds are of particular interest owing to their plentiful applications in materials science and pharmaceutical chemistry.^1,2^ Organosilanes are versatile intermediates in organic synthesis,^3^ since the C–Si bond can be readily transformed into C–O bonds and C–C bonds. Thus, various synthetic methods for obtaining organosilicon compounds have been developed, including silyl addition to carboxylate derivatives,^4^ radical-mediated C(sp^3^)–Si cross-coupling reactions,^5^ ring-opening C(sp^3^)–Si bond-forming reactions,^6^ defluorosilylation of fluoroalkenes,^7^ carbosilylation of unsaturated hydrocarbons,^8^ silylation of aldehydes^9^ and conjugate silyl addition to unsaturated carbonyl compounds^10^ and dienes,^11^*etc.* Among these available approaches to organosilicon compounds, silyl transfer from silylboranes (*e.g.*, PhMe_2_Si-Bpin)^12^ to unsaturated acceptors, especially transition metal-free organocatalytic silylation is becoming increasingly attractive to organic chemists (Scheme 1a).^13^

With the development of the Si–B bond activation,^14^ Tortosa and co-workers firstly reported an efficient and general copper(i)-catalyzed protocol for the addition of nucleophilic silicon species to *p*-quinone methides (Scheme 1b).^15^ Due to the low-abundance and high toxicity of the transition metal, the development of environmentally friendly and transition metal-free methods for chemical biology studies and pharmaceutical synthesis has been highly desirable. As part of our continuing efforts in metal-free catalytic C–Si bond-formations,^16^ we herein disclose a new entry of Cs_2_CO_3_-catalyzed transition metal-free silylative aromatization of *p*-quinone methides.

As a further benefit, the resulting benzylic silane acts as bench-stable carbanion for preparation of the corresponding carboxylic acid through addition of gaseous CO_2_ under mild reaction conditions. This synthetic protocol is very attractive not only for the utilization of CO_2_, an inexpensive and sustainable C1 source, but also for decreasing the CO_2_, the most significant long-live greenhouse gas in the atmosphere. To the best of our knowledge, the use of dibenzylic anion from a silane for the addition reaction of carbon dioxide has not previously been reported.

## Results and discussion

It is well established that water as an additive has a beneficial effect on organocatalytic silyl transfer reaction.^13,16^ Thus, we commenced the study of silylative aromatization reaction by using 4-benzylidene-2,6-di-*tert*-butylcyclohexa-2,5-dien-1-one (1a) as the model substrate, Me_2_PhSi-Bpin as the silylating reagent and water as the additive (Table 1). As reported by Tortosa,^15^ using THF as the solvent, the transition metal-free silyl transfer reactions did not take place to any appreciable extent after 24 hours (entries 1–3).

**Table 1:** Optimization of reaction conditions

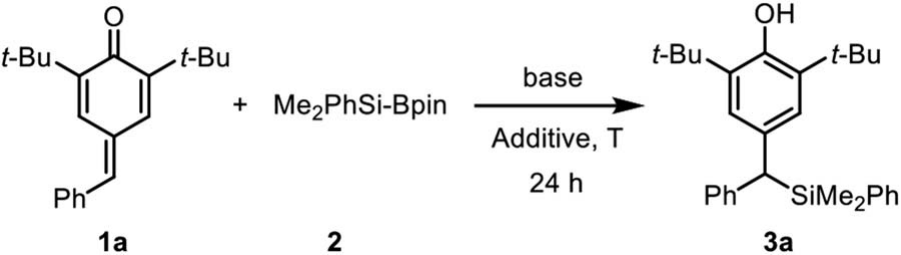				
Entry	*T*/°C	Base (mol%)	Additive (equiv.)	Yield^a^ (%)
1^b^	rt	NaO*t*-Bu (20)	MeOH (4)	—
2^c^	rt	Cs_2_CO_3_ (5)	H_2_O (1)	<5
3^c^	rt	Cs_2_CO_3_ (5)	—	<5
4	rt	Cs_2_CO_3_ (5)	H_2_O (1)	14
5	60	Cs_2_CO_3_ (5)	H_2_O (1)	55
6	60	Cs_2_CO_3_ (5)	—	47
7	70	Cs_2_CO_3_ (5)	H_2_O (1)	63
8	80	Cs_2_CO_3_ (5)	H_2_O (1)	67
9	90	Cs_2_CO_3_ (5)	H_2_O (1)	69
10	80	Cs_2_CO_3_ (5)	H_2_O (0.5)	73
11	80	Cs_2_CO_3_ (5)	H_2_O (2)	71
12	80	K_2_CO_3_ (5)	H_2_O (0.5)	70
13	80	KOSi(CH_3_)_3_ (5)	H_2_O (0.5)	64
14	80	KF (5)	H_2_O (0.5)	57
15	80	KOAc (5)	H_2_O (0.5)	67
16	80	KO*t*-Bu (5)	H_2_O (0.5)	62
17	80	DBU (5)	H_2_O (0.5)	51
18^d^	80	Cs_2_CO_3_ (5)	H_2_O (0.5)	95

a Yields were determined by ^1^H NMR analysis.

b Reaction conditions: 1a (0.2 mmol), 2 (0.22 mmol), NaO*t*-Bu (20 mol%), MeOH (0.8 mmol), THF (0.1 M), 12 h.

c Reaction conditions: 1a (0.1 mmol), 2 (0.15 mmol), Cs_2_CO_3_ (5 mol%), THF (0.1 M).

d 2 (0.25 mmol) used.

Disappointed by these results, we moved attention to our previous research findings,^17^ in which azidative aromatization of *p*-quinone methides was achieved with cesium carbonate as a catalyst and water as an effective additive under transition metal and solvent free conditions. We anticipated a solvent-free reaction condition might be a viable option so as to compensate for the lack of reactivity. We therefore decided to run this reaction under solvent-free conditions, in an attempt to accelerate reaction rate by means of efficient and environmentally friendly procedures. Although the silylation was sluggish, the desired product 3a was obtained in 14% yield after 24 hours at room temperature (entry 4). The low yield could be attribute to the low reaction temperature because most of the substrate 1a as a yellow solid remained unchanged.

**Scheme 2:**
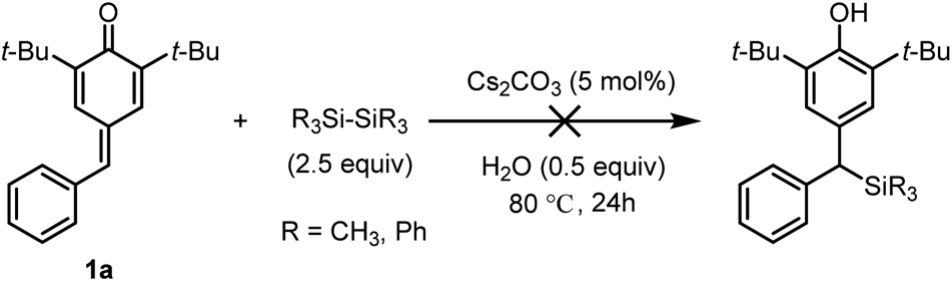
Screening of disilanes.

In line with our expectation, the silyl addition reaction proceeded smoothly providing the product 3a in 55% yield by heating the reaction mixture to 60 °C for 24 h (entry 5). As a comparative illustration, the efficiency of water to promote addition was evident as a 47% yield was obtained in the absence of water (entry 6). Encouragingly, the yield was increased to 67% by raising the reaction temperature to 80 °C (entries 7–8). However, a further increase in the reaction temperature proved to be almost ineffective (entry 9), so 80 °C was chosen as the suitable reaction temperature for further investigation. Again, the impact of water at 80 °C was tested, and 0.5 equiv. of water was found to be more competitive (entries 10–11). To improve the yield, we then examined the influence of the base with a particular emphasis on the effect of escorting counterion. It was found that carbonate was superior to trimethylsilanolate, fluoride, acetate, *tert*-butoxide as well as DBU, thus indicating that these variables was incompetent to improve the yield (entries 12–17). Notably, unreacted substrate 1a was observed in the ^1^H NMR spectrum of the crude product, but the silylating reagent (Me_2_PhSi-Bpin) could not be found in all the reaction mixtures at higher reaction temperature (entries 5–17). These results showed that the adding quantity of Me_2_PhSi-Bpin did not meet the needs of the reaction due to its high reactivity and low stability, so more silaborane reagent might be needed to fill in the gaps resulted from thermal decomposition. As expected, complete conversion of 1a was achieved in 95% yield by increasing the amount of 2 from 1.5 to 2.5 equiv. (entry 18). Furthermore, two different disilanes were also tested under the optimized reaction conditions (Scheme 2). Unfortunately, both hexamethyldisilane and hexaphenyldisilane failed to give the desired products.

With the optimized reaction conditions in hand, the substrate scope of the silylative aromatization of *p*-quinone methides was investigated (Table 2). A wide range of *p*-quinone methides bearing electron-deficient groups at different positions on the phenyl ring were well compatible with the reaction conditions, and afforded the silyl transfer products in 79–96% yields (3h–3l). However, the substrate containing an electron-donating group at the benzene ring such as methyl and methoxy proved to be less reactive, so the reaction time had to be prolonged to 48 h (3b–3g). Similarly, the *p*-quinone methide having a naphthalene ring was not a good substrate and provided the corresponding product 3m in lower yield (78%). It was found that an electron-rich heteroaromatic ring such as thiophene was also amenable to the aforementioned protocol, and gave the desired product 3n in 84% yield. In contrast, an electron-deficient heteroaromatic ring like pyridine was very suitable for the reaction and the silyl addition product 3o was obtained in 83% yield, even though the reaction was conducted at 40 °C for 24 h. In line with our expectation, the scope was also extended to aliphatic-substituted *p*-quinone methide, which furnished the desired product in good yield (83%).

**Table 2:** Substrate scope^a^^,^^b^

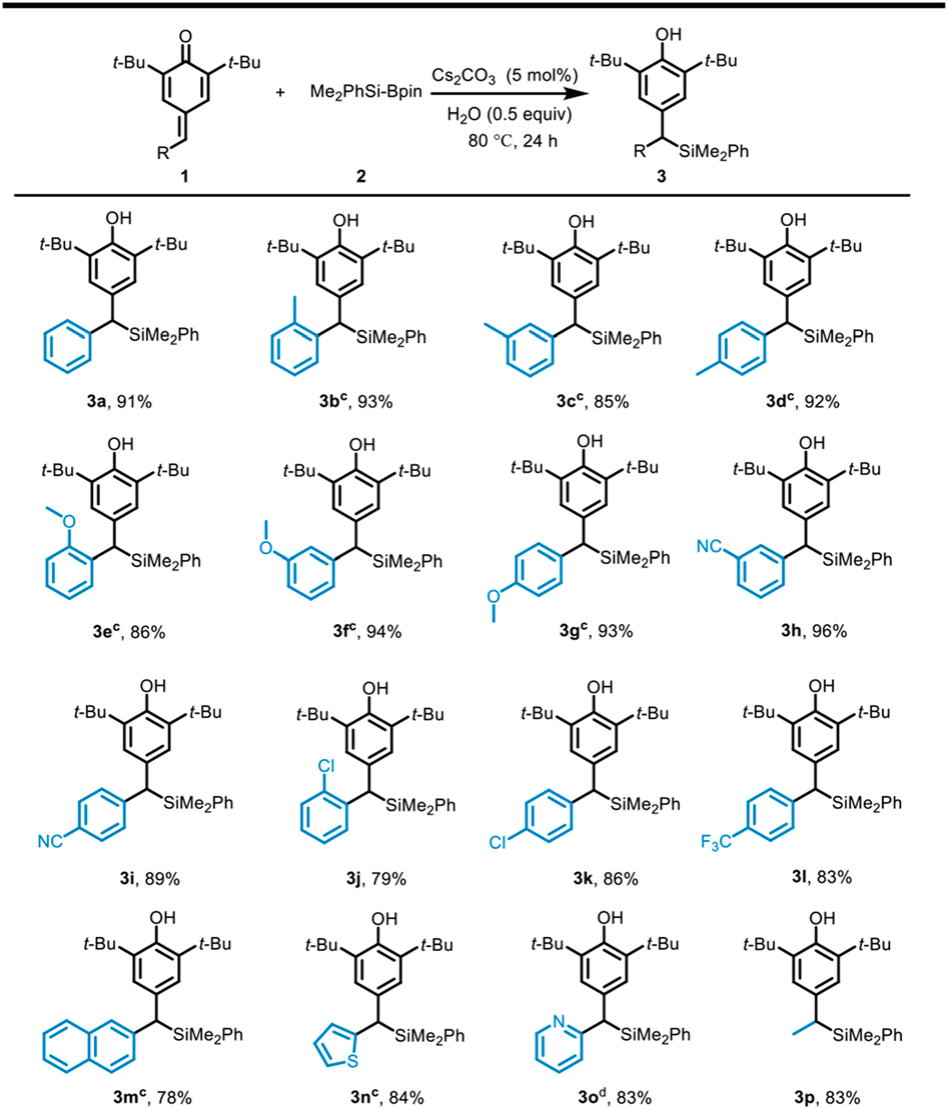

a Reaction conditions: 1 (0.2 mmol), 2 (2.5 equiv.), Cs_2_CO_3_ (5 mol%), H_2_O (0.5 equiv.), 80 °C, 24 h.

b Yield of isolated 3.

c Reaction time was 48 h.

d 40 °C, 24 h.

**Scheme 3:**
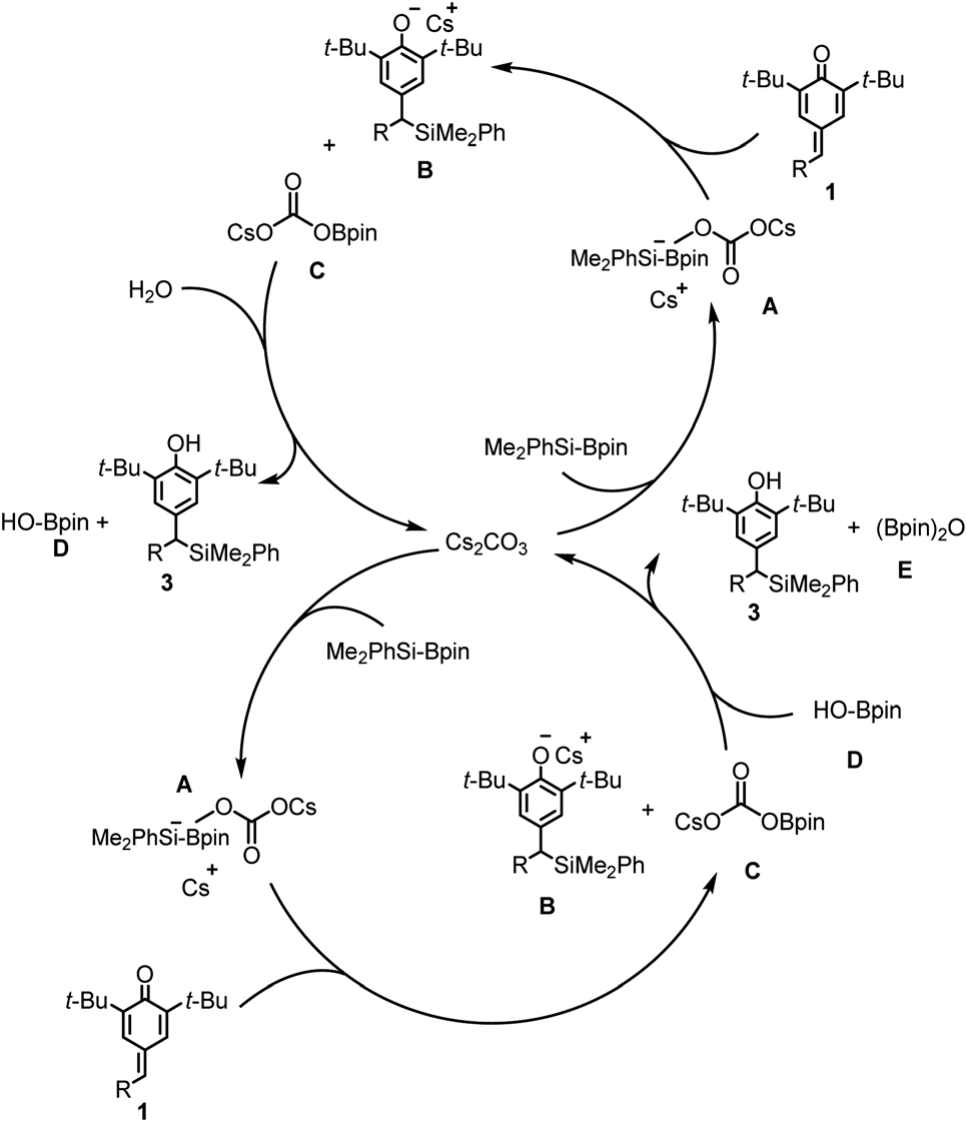
Proposed catalytic cycle.

A tentative catalytic cycle for the silylative aromatization of *p*-quinone methides is depicted in Scheme 3. The formation of benzylic silanes is assumed to involve an active complex A, the adduct of Cs_2_CO_3_ with Me_2_PhSi-Bpin, which undergoes addition of the exocyclic double bond of *p*-quinone methide 1 to generate the intermediates B and C. The intermediates B is then protonated by H_2_O to release the final product 3, and by-product D which involves another circle to form E. Such a catalytic cycle explains why the mole ratio between 1 and H_2_O is 2 : 1 under the optimized reaction conditions.

**Scheme 4:**
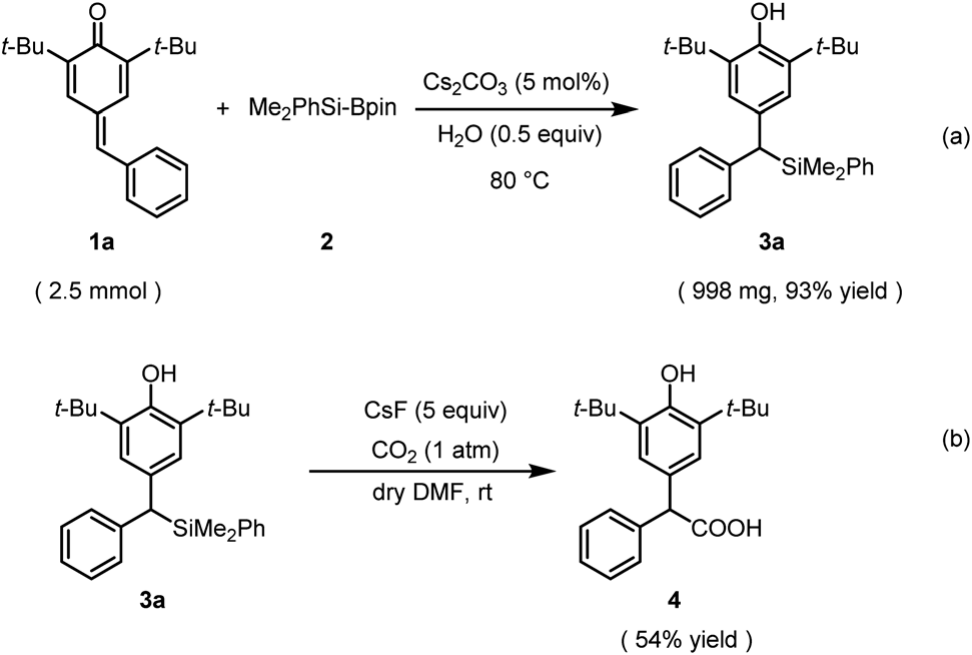
Scale-up experiment and synthetic application of compound 3a.

To demonstrate the synthetic utilities of this catalytic process, a gram-scale reaction was performed under the standard reaction conditions. As anticipated, the reaction was completed in 36 h and afforded the corresponding benzylic silane 3a in 93% yield (Scheme 4a). Furthermore, organosilane 3a as a bench-stable carbanion enabled the incorporation of gaseous CO_2_ into a value-added carboxylic acid under mild reaction conditions (Scheme 4b).

## Experimental

### General information

Commercially available reagents were used without further purification unless otherwise noted. Solvents were reagent grade and purified by standard techniques. Purification of the reaction products was carried out by chromatography on silica gel (200–300 mesh). ^1^H NMR and ^13^C NMR spectra were recorded in CDCl_3_ on a Bruker AVANCE-400 or Bruker AVANCE-500 spectrometer at 298 K. Mass spectra were recorded on an Agilent Technologies 6510 Q-Tof LC/MS. All melting points were recorded on a melting point apparatus and were uncorrected. All reactions were monitored by TLC with silica gel-coated plates and visualized with a UV light at 254 nm.

### General procedure for synthesis of dibenzylic silanes (3)

To an oven-dried vial was charged 1.8 µl H_2_O, Cs_2_CO_3_ (3.2 mg, 0.01 mmol), the indicated *para*-quinone methide 1^18^ (0.2 mmol) and a stir bar. Me_2_PhSi-Bpin (145 µl, 0.5 mmol) was taken under an N_2_ atmosphere and added into the vial by syringe. After the mixture was stirred under 80 °C for 24 h or 48 h, the mixture was diluted by petroleum ether and a few drops of CH_3_COOH was added. The solvent was removed in vacuum and the crude product was purified by flash column chromatography (petroleum ether/chloroform = 5 : 1–1 : 2) to afford the corresponding product 3.

### General procedure for the gram-scale synthesis of dibenzylic silanes (3a)

To an oven-dried vial was charged 22.5 µl H_2_O, Cs_2_CO_3_ (40.8 mg, 0.125 mmol), the indicated *para*-quinone methide 1a (0.74 g, 2.5 mmol) and a stir bar. Me_2_PhSi-Bpin (1.8 ml, 6.25 mmol) was taken under an N_2_ atmosphere and added into the vial by syringe. After the mixture was stirred under 80 °C for 36 h, the mixture was diluted by petroleum ether and a few drops of CH_3_COOH was added. The solvent was removed in vacuum and the crude product was purified by flash column chromatography (petroleum ether/chloroform = 5 : 1) to afford the corresponding product 3a (998 mg, 93% yield).

### General procedure for preparing the compound acid^19^ (4)

An oven-dried two-necked vial was charged with CsF (151.9 mg, 1.0 mmol, 5.0 equiv.) and a stir bar, and then dried with a heat gun for 2 min under vacuum (about 5 mm Hg at *ca.* 400 °C). After the displacement with CO_2_ gas, 3a (86 mg, 0.2 mmol, 1.0 equiv.) dissolved in dry DMF (4.0 ml) was added to the vial. The resulting reaction mixture was stirred at rt for 48 h under CO_2_ atmosphere (1 atm, balloon). Water was added to the reaction mixture followed by the acidification (pH = *ca.* 2) using 1 M HCl. The mixture was extracted with dichloromethane for 3 times, then the organic layers were combined and washed with water for 3 times, finally dried over anhydrous MgSO_4_. The solvent was then removed under reduced pressure and the residue was purified by flash column chromatography (petroleum ether/ethyl acetate = 10 : 1–5 : 1) to afford the corresponding product 4 (37 mg, 54% yield).

## Conclusions

In conclusion, we have developed a base-mediated silylation reaction which can be effectively performed on a gram scale. The present study exhibits that the silylative aromatization of a wide array of *p*-quinone methides is achieved by Cs_2_CO_3_ catalyst without the needs for any harmful organic solvents and an air- and moisture-sensitive copper(i) salt. To the best of our knowledge, this is the first example which enables the silyl transfer from silylborane (*e.g.*, PhMe_2_Si-Bpin) to unsaturated acceptors under transition metal and solvent-free conditions. Furthermore, carboxylation of the as-obtained organosilane with gaseous CO_2_ provides a new synthetic protocol for preparation of a value-added carboxylic acid. The study of an asymmetric variant of this silylative aromatization is in progress.

## Conflicts of interest

There are no conflicts to declare.

## Supplementary Material

RA-011-D1RA03193G-s001
