# Supplementary material for: Silylative aromatization of p-quinone methides under metal and solvent free conditions
Source: RSC Adv. 2021 May 18;11(29):17860–4. doi: 10.1039/d1ra03193g (PMC9033227; doi:10.1039/d1ra03193g)

Electronic Supplementary Material (ESI) for RSC Advances.
This journal is © The Royal Society of Chemistry 2021

Cesium Carbonate Catalyzed Silylative Aromatization of *p*-Quinone Methides under Solvent-Free Conditions

Tingting Li,[a] Yuzhu Wu,[a] Wenzeng Duan,*[b] and Yudao Ma*[a]

[a] *Department of Chemistry, Shandong University, Shanda South Road No. 27, Jinan 250100, P. R. China.*
[b] *School of Chemistry and Chemical Engineering, Liaocheng University, Liaocheng 252000, P. R. China.*

Table of contents

1, General Information········· 2

2, Experimental Section········· 2

3, References········· 9

4, Copies of NMR spectra········· 10

* Corresponding authors.

E-mail addresses: ydma@sdu.edu.cn (Y. Ma);

duanwenzeng@lcu.edu.cn (W. Duan).

1, General Information

Commercially available reagents were used without further purification unless otherwise noted. Solvents were reagent grade and purified by standard techniques. Purification of the reaction products was carried out by chromatography on silicagl (200-300 mesh). ^1H NMR and ^{13}C NMR spectra were recorded on a Bruker AVANCE-400 or Bruker AVANCE-500 spectrometer at 298 K. Chemical shifts were reported as δ values in ppm and the tetramethylsilane was used as an internal standard in $CDCl_3$. Data are reported as (s = singlet, d = doublet, dd = doublet of doublet, t = triplet, dt = doublet of triplet, td = triplet of doublet, q = quartet, qd = quartet of doublet, m = multiplet, brs = broad singlet). Mass spectra were recorded on an Agilent Technologies 6510 Q-Tof LC/MS. All melting points were recorded on a melting point apparatus and were uncorrected. All reactions were monitored by TLC with silica gel-coated plates and visualized with a UV light at 254 nm.

2, Experimental Section

General procedure for the synthesis of dibenzylic silanes 3

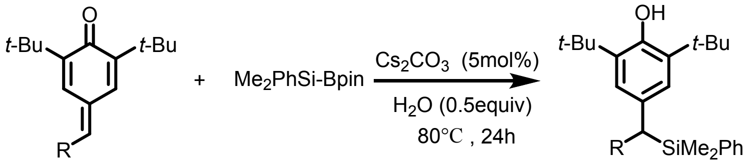

1 **2** **3**

To an oven-dried vial was charged 1.8 µl H_2O, Cs_2CO_3 (3.2 mg, 0.01 mmol), the indicated *para*-quinone methide **1**[1] (0.2 mmol) and a stir bar. Me_2PhSi-Bpin (145 µl, 0.5 mmol) was taken under an N_2 atmosphere and added into the vial by syringe. After the mixture was stirred under 80 °C for 24 h or 48 h, the mixture was diluted by petroleum ether and a few drops of CH_3COOH was added. The solvent was removed in vacuum and the crude product was purified by flash column chromatography (petroleum ether/chloroform = 5:1-1:2) to afford the corresponding product **3**.

2,6-di(*tert*-butyl)-4-((dimethyl(phenyl)silyl)(phenyl)methyl)phenol (3a):

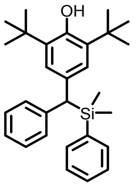

From **1a**, following the general procedure described above, compound **3a** was obtained in 91% yield as a colorless oil.

^1H NMR (500MHz, $CDCl_3$): δ 7.40-7.35 (m, 1H), 7.33-7.24 (m, 6H), 7.21-7.14 (m, 3H), 6.92 (s, 2H), 3.68 (s, 1H), 1.39 (s, 18H), 0.33 (s, 3H), 0.31 (s, 3H).

^{13}C NMR (125MHz, $CDCl_3$): δ 151.5, 143.2, 138.1, 135.5, 134.7, 132.5, 129.1, 129.1, 128.3, 127.6, 125.6, 125.1, 45.7, 34.5, 30.5, -3.0, -3.3.

HRMS (ESI) m/z calcd for $C_{29}H_{39}OSi$ $[M + H]^+$431.2765, found 431.2744.

2,6-di(*tert*-butyl)-4-((dimethyl(phenyl)silyl)(*o*-tolyl)methyl)phenol (3b):

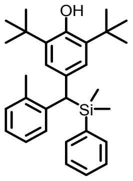

From **1b**, following the general procedure described above, compound **3b** was obtained in 93% yield as a colorless oil.

^{1}H NMR (500MHz, $CDCl_3$): δ 7.38-7.34 (m, 1H), 7.32-7.26 (m, 4H), 7.14 (t, J = 7.5Hz, 1H), 7.01-6.95 (m, 3H), 6.89 (s, 2H), 4.95 (s, 1H), 3.60 (s, 1H), 2.29 (s, 3H), 1.37 (s, 18H), 0.30 (s, 3H), 0.28 (s, 3H).

^{13}C NMR (125MHz, $CDCl_3$): δ 151.4, 143.0, 138.2, 137.6, 135.4, 134.7, 132.5, 130.2, 129.1, 128.1, 127.6, 126.0, 125.9, 125.5, 45.6, 34.5, 30.5, 21.7, -2.9, -3.3.

HRMS (ESI) m/z calcd for $C_{30}H_{41}OSi$ [M + H] $^+$445.2921, found 445.2831.

2,6-di(*tert*-butyl)-4-((dimethyl(phenyl)silyl)(*m*-tolyl)methyl)phenol (3c):

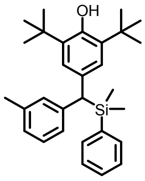

From **1c**, following the general procedure described above, compound **3c** was obtained in 91% yield as a colorless oil.

^{1}H NMR (500MHz, $CDCl_3$): δ 7.38-7.34 (m, 1H), 7.32-7.28 (m, 5H), 7.15-7.11 (m, 2H), 7.08-7.04 (m, 1H), 6.84 (s, 2H), 4.94 (s, 1H), 3.85 (s, 1H), 2.28 (s, 3H), 1.34 (s, 18H), 0.34 (s, 3H), 0.30 (s, 3H).

^{13}C NMR (100MHz, $CDCl_3$): δ 151.3, 141.6, 138.6, 136.7, 135.2, 134.6, 132.1, 130.7, 129.6, 129.1, 127.7, 126.7, 125.5, 125.3, 40.3, 34.4, 30.5, 20.8, -2.4, -3.4.

HRMS (ESI) m/z calcd for $C_{30}H_{41}OSi$ [M + H] $^+$445.2921, found 445.2852.

2,6-di(*tert*-butyl)-4-((dimethyl(phenyl)silyl)(*p*-tolyl)methyl)phenol (3d):

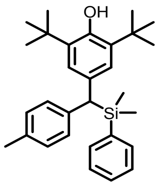

From **1d**, following the general procedure described above, compound **3d** was obtained in 92% yield as a colorless oil.

^{1}H NMR (500MHz, $CDCl_3$): δ 7.40-7.36 (m, 1H), 7.35-7.31 (m, 4H), 7.13-7.07 (m, 4H), 6.92 (s, 2H), 4.98 (s, 1H), 3.65 (s, 1H), 2.35 (s, 3H), 1.40 (s, 18H), 0.34 (s, 3H), 0.31 (s, 3H);

^{13}C NMR (100MHz, $CDCl_3$): δ 151.4, 140.1, 138.3, 135.4, 134.7, 134.4, 132.7, 129.1, 129.0, 129.0, 127.6, 125.5, 45.1, 34.5, 30.5, 21.1, -2.8, -3.2.

HRMS (ESI) m/z calcd for $C_{30}H_{41}OSi$ [M + H] $^+$445.2921, found 445.2848.

2,6-di(*tert*-butyl)-4-((dimethyl(phenyl)silyl)(2-methoxyphenyl)methyl)phenol (3e):

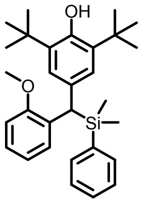

From **1e**, following the general procedure described above, compound **3e** was obtained in 86% yield as a light yellow oil.

^{1}H NMR (500MHz, $CDCl_3$): δ 7.38-7.34 (m, 1H), 7.34-7.30 (m, 4H), 7.27-7.24 (m, 1H), 7.17-7.13 (m, 1H), 6.95 (s, 2H), 6.94-6.88 (m, 1H), 6.85 (d, J = 8.15Hz, 1H), 4.96 (s, 1H), 4.24 (s, 1H), 3.75 (s, 3H), 1.39 (s, 18H), 0.31 (s, 3H), 0.29 (s, 3H).

^{13}C NMR (100MHz, $CDCl_3$): δ 156.8, 151.3, 139.2, 135.2, 134.5, 132.7, 131.9, 130.3, 128.8, 126.2, 125.8, 120.4, 110.5, 55.2, 36.7, 34.5, 30.5, -2.6, -3.3.

HRMS (ESI) m/z calcd for $C_{30}H_{41}O_2Si$ $[M + H]^+$ 461.2870, found 461.2792.

2,6-di(*tert*-butyl)-4-((dimethyl(phenyl)silyl)(3-methoxyphenyl)methyl)phenol (3f):

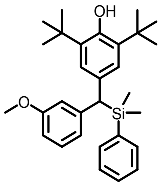

From **1f**, following the general procedure described above, compound **3f** was obtained in 94% yield as a light yellow oil.

^{1}H NMR (500MHz, $CDCl_3$): δ 7.5-7.35 (m, 1H), 7.34-7.29 (m, 4H), 7.20-7.15 (m, 1H), 6.94 (s, 2H), 6.81 (d, J = 7.7Hz, 1H), 6.73-6.70 (m, 2H), 5.00 (s, 1H), 3.71 (s, 3H), 3.65 (s, 1H), 1.40 (s, 18H), 0.33 (s, 3H), 0.32 (s, 3H).

^{13}C NMR (100MHz, $CDCl_3$): δ 159.5, 151.5, 144.7, 138.1, 135.4, 134.7, 132.3, 129.2, 129.1, 127.6, 125.6, 121.5, 114.2, 111.1, 55.1, 45.9, 34.5, 30.5, -3.0, -3.2.

HRMS (ESI) m/z calcd for $C_{30}H_{41}O_2Si$ $[M + H]^+$ 461.2870, found 461.2867.

2,6-di(*tert*-butyl)-4-((dimethyl(phenyl)silyl)(4-methoxyphenyl)methyl)phenol (3g):

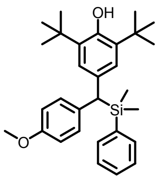

From **1g**, following the general procedure described above, compound **3g** was obtained in 92% yield as a light yellow oil.

^{1}H NMR (500MHz, $CDCl_3$): δ 7.41-7.36 (m, 1H), 7.34-7.29 (m, 4H), 7.15-7.11 (m, 2H), 6.90 (s, 2H), 6.85-6.81 (m, 2H), 4.98 (s, 1H), 3.82 (s, 3H), 3.63 (s, 1H), 1.39 (s, 18H), 0.33 (s, 3H), 0.31 (s, 3H).

^{13}C NMR (100MHz, $CDCl_3$): δ 157.3, 151.4, 138.2, 135.4, 135.3, 134.7, 132.8, 130.0, 129.1, 128.6, 125.4, 113.7, 55.3, 44.5, 34.5, 30.5, -2.8, -3.3.

HRMS (ESI) m/z calcd for $C_{30}H_{41}O_2Si$ $[M + H]^+$ 461.2870, found 461.2798.

2,6-di(*tert*-butyl)-4-((dimethyl(phenyl)silyl)(3-cyanophenyl)methyl)phenol (3h):

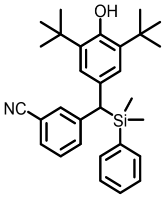

From **1h**, following the general procedure described above, compound **3h** was obtained in 96% yield as a colorless oil.

^{1}H NMR (500MHz, $CDCl_3$): δ 7.44-7.41 (m,1H), 7.40-7.29 (m, 7H), 7.25-7.24 (m, 1H), 6.88 (s, 2H), 5.06 (s, 1H), 3.69 (s, 1H), 1.39 (s, 18H), 0.32-0.31 (d, *J* = 2.35Hz, 6H).

^{13}C NMR (100MHz, $CDCl_3$): δ 151.9, 145.0, 136.9, 135.9, 134.5, 133.2, 132.2, 131.1, 129.6, 128.9, 128.8, 127.8, 125.7, 119.4, 112.2, 45.5, 34.5, 30.4, -3.2, -3.3.

HRMS (ESI) m/z calcd for $C_{30}H_{38}NOSi$ [M + H]$^+$456.2717, found 456.2711.

2,6-di(*tert*-butyl)-4-((dimethyl(phenyl)silyl)(4-cyanophenyl)methyl)phenol (3i):

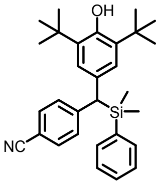

From **1i**, following the general procedure described above, compound **3i** was obtained in 89% yield as a colorless oil.

^{1}H NMR (500MHz, $CDCl_3$): δ 7.51-7.47 (m, 2H), 7.40-7.36 (m, 1H), 7.32-7.28 (m, 2H), 7.25-7.22 (m, 2H), 7.19-7.17 (m, 2H), 6.88 (s, 2H), 5.04 (s, 1H), 3.72 (s, 1H), 1.37 (s, 18H), 0.30-0.29 (d, *J* = 2.05Hz, 6H).

^{13}C NMR (100MHz, $CDCl_3$): δ 152.0, 149.5, 136.9, 135.9, 134.5, 131.2, 131.0, 129.5, 129.3, 127.8, 125.8, 119.5, 108.6, 46.5, 34.5, 30.5, -3.1, -3.3.

HRMS (ESI) m/z calcd for $C_{30}H_{38}NOSi$ [M + H]$^+$456.2717, found 456.2717.

2,6-di(*tert*-butyl)-4-((2-chlorophenyl)(dimethyl(phenyl)silyl)methyl)phenol (3j):

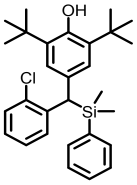

From **1j**, following the general procedure described above, compound **3j** was obtained in 79% yield as a colorless oil.

^{1}H NMR (500MHz, $CDCl_3$): δ 7.39-7.30 (m, 7H), 7.18 (td, *J* = 7.45, 1.3Hz, 1H), 7.09 (td, *J* = 7.8, 1.65Hz, 1H), 6.91 (s, 2H), 4.99 (s, 1H), 4.40 (s, 1H), 1.37 (s, 18H), 0.36 (s, 3H), 0.31 (s, 3H).

^{13}C NMR (125MHz, $CDCl_3$): δ 151.6, 141.1, 137.9, 135.4, 134.7, 134.6, 131.5, 130.6, 129.9, 129.2, 127.7, 126.5, 125.8, 40.1, 34.5, 30.5, -2.6, -3.6.

HRMS (ESI) m/z calcd for $C_{29}H_{38}ClOSi$ [M + H]$^+$ 465.2375, found 465.2326.

2,6-di(*tert*-butyl)-4-((4-chlorophenyl)(dimethyl(phenyl)silyl)methyl)phenol (3k):

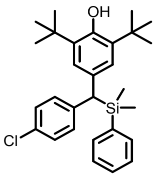

From **1k**, following the general procedure described above, compound **3k** was obtained in 86% yield as a colorless oil.

^{1}H NMR (500MHz, $CDCl_3$): δ 7.41-7.37 (m, 1H), 7.34-7.27 (m, 4H), 7.24-7.21 (m, 2H), 7.11-7.08 (m, 2H), 6.89 (s, 2H), 5.02 (s, 1H), 3.65 (s, 1H), 1.39 (s, 18H), 0.32 (d, J = 4.35Hz, 6H).

^{13}C NMR (125MHz, $CDCl_3$): δ 151.6, 141.8, 137.6, 135.6, 134.6, 132.0, 130.8, 130.3, 129.3, 128.3, 127.7, 125.5, 45.0, 34.5, 30.5, -3.0, -3.2.

HRMS (ESI) m/z calcd for $C_{29}H_{38}ClOSi$ [M + H]$^+$ 465.2375, found 465.2320.

2,6-di(*tert*-butyl)-4-((dimethyl(phenyl)silyl)(4-(trifluoromethyl)phenyl)methyl)phenol (3l)：

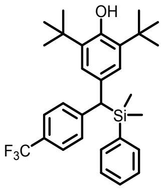

From **1l**, following the general procedure described above, compound **3l** was obtained in 83% yield as a colorless oil.

^{1}H NMR (500MHz, $CDCl_3$): δ 7.49 (d, J = 8.2Hz, 2H), 7.40-7.36 (m, 1H), 7.33-7.29 (m, 2H), 7.28-7.23 (m, 4H), 6.90 (s, 2H), 5.02 (s, 1H), 3.73 (s, 1H), 1.38 (s, 18H), 0.30 (d, J = 4.3Hz, 6H).

^{13}C NMR (100MHz, $CDCl_3$): δ 151.8, 147.7 (q, $J_{C\text{-}F}$ = 1.4 Hz), 137.3, 135.7, 134.6, 131.5, 129.4, 129.0, 127.8, 127.3 (q, $J_{C\text{-}F}$ = 32.07 Hz), 125.7, 125.2 (q, $J_{C\text{-}F}$ = 4.67 Hz), 124.7 (q, $J_{C\text{-}F}$ = 269.94 Hz), 46.0, 34.5, 30.5, -3.1, -3.2.

HRMS (ESI) m/z calcd for $C_{30}H_{38}F_3OSi$ [M + H]$^+$ 499.2639, found 499.2604.

2,6-di(*tert*-butyl)-4-((dimethyl(phenyl)silyl)(naphthalen-2-yl)methyl)phenol (3m):

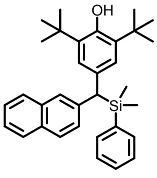

From **1m**, following the general procedure described above, compound **3m** was obtained in 78% yield as a light yellow oil.

^{1}H NMR (500MHz, $CDCl_3$): δ 7.83 (d, J = 7.8Hz, 1H), 7.77-7.73 (m, 2H), 7.65 (s, 1H), 7.49-7.40 (m, 3H), 7.37-7.32 (m, 5H), 7.01 (s, 2H), 3.88 (s, 1H), 1,41 (s, 18H), 0.38 (s, 3H), 0.35 (s, 3H).

^{13}C NMR (125MHz, $CDCl_3$): δ 151.6, 140.9, 138.0, 135.5, 134.7, 133.8, 132.4, 131.7, 129.2, 128.5, 127.7(2C), 127.6, 126.9, 125.8, 125.7, 125.0, 45.7, 34.5, 30.5, -2.8, -3.2.

HRMS (ESI) m/z calcd for $C_{33}H_{41}OSi$ [M + H]$^+$ 481.2921, found 481.2835.

2,6-di(*tert*-butyl)-4-((dimethyl(phenyl)silyl)(thiophen-2-yl)methyl)phenol (3n):

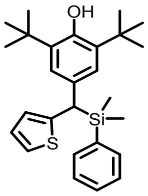

From **1n**, following the general procedure described above, compound **3n** was obtained in 84% yield as a colorless oil.

^{1}H NMR (500MHz, $CDCl_3$): δ 7.39-7.35 (m, 1H), 7.32-7.26 (m, 4H), 7.07 (dd, J = 5.15, 0.9Hz, 1H), 6.94-6.91 (m, 1H), 6.89 (s, 2H), 6.78 (d, J = 3.4Hz, 1H), 4.98 (s, 1H), 3.93 (s, 1H), 1.38 (s, 18H), 0.34 (s, 3H), 0.33 (s, 3H).

^{13}C NMR (100MHz, $CDCl_3$): δ 151.6, 146.2, 137.2, 135.4, 134.7, 131.7, 129.3, 127.6, 126.8, 125.0, 124.2, 122.3, 40.4, 34.5, 30.5, -3.5, -3.9.

HRMS (ESI) m/z calcd for $C_{27}H_{37}SOSi$ $[M + H]^+$ 437.2329, found 437.2330.

2,6-di(*tert*-butyl)-4-((dimethyl(phenyl)silyl)(pyridin-2-yl)methyl)phenol (3o):

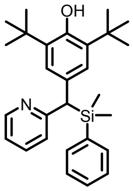

From **1o**, following the general procedure described above, compound **3o** was obtained in 83% yield as a colorless oil.

^{1}H NMR (500MHz, $CDCl_3$): δ 8.55-8.53 (m, 1H), 7.48 (td, J = 7.7, 1,7Hz, 1H), 7.33-7.28 (m, 1H), 7.26-7.21 (m, 4H), 7.07 (d, J = 7.9Hz, 1H), 7.04-7.00 (m, 1H), 6.99 (s, 2H), 4.94 (s, 1H), 3.81 (s, 1H), 1.33 (s, 18H), 0.33 (s, 3H), 0.27 (s, 3H).

^{13}C NMR (125MHz, $CDCl_3$): δ 163.4, 151.5, 135.3, 134.5, 128.9, 127.5, 125.5, 123.6, 120.2, 48.1, 34.4, 30.5, -3.3, -3.6.

HRMS (ESI) m/z calcd for $C_{28}H_{38}OSi$ $[M + H]^+$ 432.2717, found 432.2729.

2,6-di(*tert*-butyl)-4-(1-(dimethyl(phenyl)silyl)ethyl)phenol (3p):

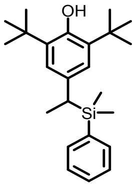

From **1p**, following the general procedure described above, compound **3p** was obtained in 83% yield as a colorless oil.

^{1}H NMR (500MHz, $CDCl_3$): δ 7.40-7.35 (m, 1H), 7.35-7.32 (m, 4H), 6.69 (s, 2H), 2.35-2.29 (m, 1H), 1.40 (s, 18H), 1.37 (d, J = 7.55Hz, 3H), 0.27 (s, 3H), 0.24 (s, 3H).

^{13}C NMR (125MHz, $CDCl_3$): δ 150.9, 138.0, 135.2, 135.0, 134.4, 128.9, 127.6, 123.9, 34.4, 30.5, 29.1, 15.1, -4.7, -4.8.

HRMS (ESI) m/z calcd for $C_{24}H_{37}OSi$ $[M + H]^+$ 369.2608, found 369.2542.

General procedure for the gram-scale synthesis of dibenzylic silanes 3a:

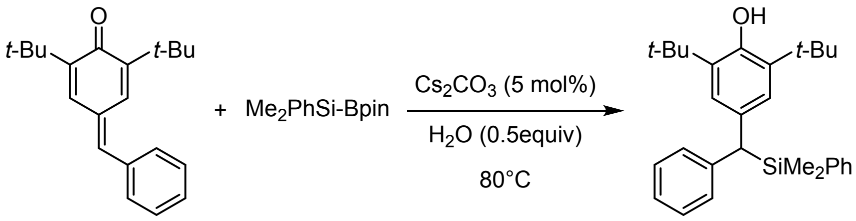

1a **2** **3a**

To an oven-dried vial was charged 22.5 µl H_2O, Cs_2CO_3 (40.8 mg, 0.125 mmol), the indicated *para*-Quinone methide **1a** (0.74 g, 2.5 mmol) and a stir bar. Me_2PhSi-Bpin (1.8 ml, 6.25 mmol) was taken under an N_2 atmosphere and added into the vial by syringe. After the mixture was stirred under 80 °C for 36 h, the mixture was diluted by petroleum ether and a few drops of CH_3COOH was added. The solvent was removed in vacuum and the crude product was purified by flash column chromatography (petroleum ether/chloroform = 5:1) to afford the corresponding product **3a** (998 mg, 93% yield).

General procedure for preparing the carboxylic acid 4[2]

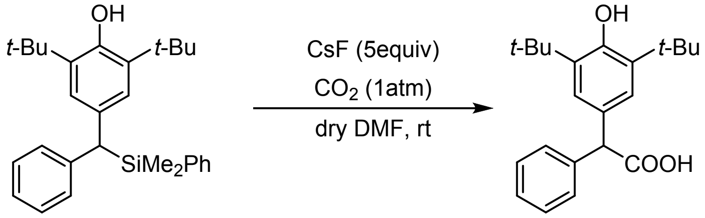

3a **4**

An oven-dried two-necked vial was charged with CsF (151.9 mg, 1.0 mmol, 5 eqiuv) and a stir bar, and then dried with a heat gun for 2 min under vacuum (＜5 mm Hg at ca. 400℃). After the displacement with CO_2 gas, **3a** (86 mg, 0.2 mmol, 1.0 equiv) dissolved in dry DMF (4.0 ml) was added to the vial. The resulting reaction mixture was stirred at r.t. for 48 h under CO_2 atmosphere (1 atm, balloon). Water was added to the reaction mixture followed by the acidification (pH = ca.2) using 1M HCl. The product was extracted with dichloromethane for 3 times, and the organic layers were combined, washed with water for 3 times and brine, and dried over anhydrous MgSO4. The solvent was then removed under reduced pressure and the residue was purified by flash column chromatography (petroleum ether/ethyl acetate = 10:1-5:1) to afford the corresponding product **4**.

2-(3,5-di(*tert*-butyl)-4-hydroxyphenyl)-2-phenylacetic acid (4)：

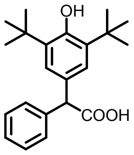

From **3a**, following the general procedure described above, compound **4** was obtained in 54% yield as a white solid; mp 162-164 ℃.

^{1}H NMR (500MHz, $CDCl_3$): δ 7.38-7.31 (m, 4H), 7.29-7.26 (m, 1H), 7.16 (s, 2H), 5.30 (s, 1H), 5.18 (s, 1H), 4.96 (s, 1H), 1.41 (s, 18H).

^{13}C NMR (100MHz, $CDCl_3$): δ 179.1, 153.4, 138.7, 136.1, 128.8, 128.7, 128.5, 127.4, 125.6, 57.1, 34.6, 30.4.

HRMS (ESI) m/z calcd for $C_{22}H_{29}O_3$ $[M + H]^+$ 341.2111, found 341.2107.

3. References

1. L. Roiser and M. Waser, *Org. Lett.*, 2017, **19**, 2338.
2. T. Mita, J. Chen, M. Sugawara and Y. Sato, *Org. Lett.*, 2012, **14**, 6202; Y. Xiao, C. Yue, P. Chen and Y. Chen, *Org. Lett.*, 2014, **16**, 3208

4.Copies of NMR spectra

^{1}H NMR Spectrum of compound 3a

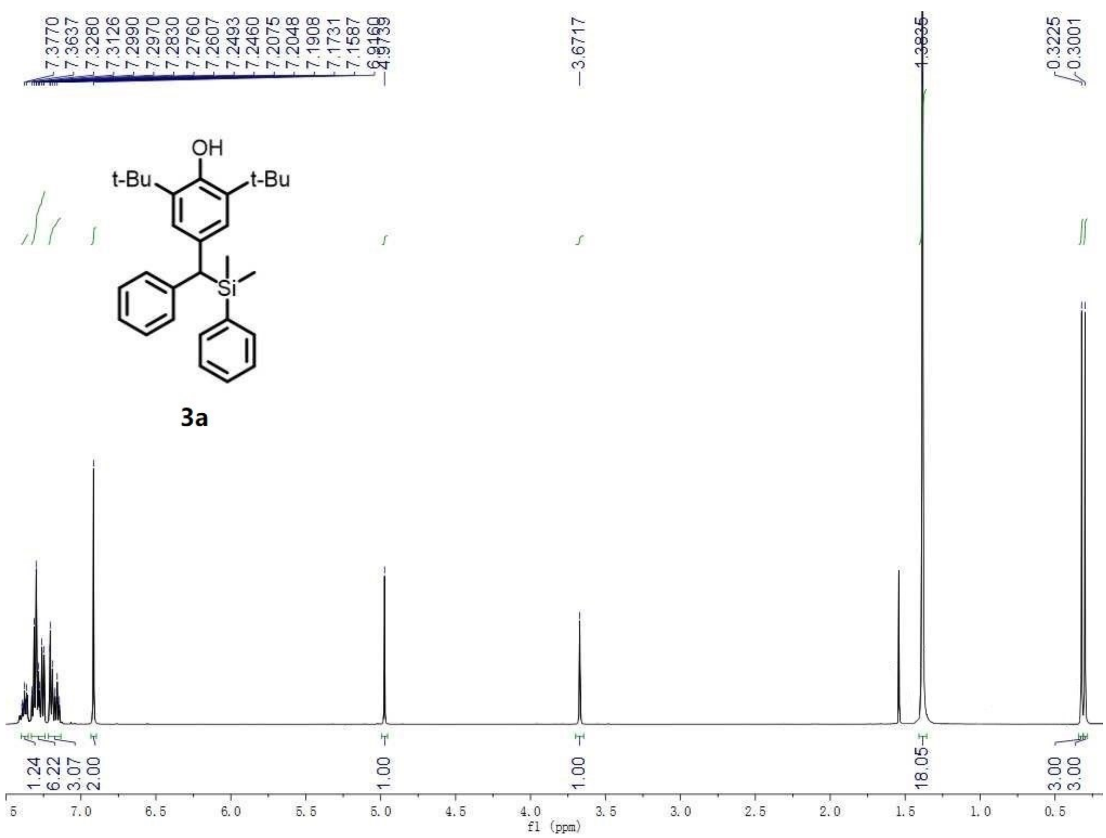

^{13}C NMR Spectrum of compound 3a

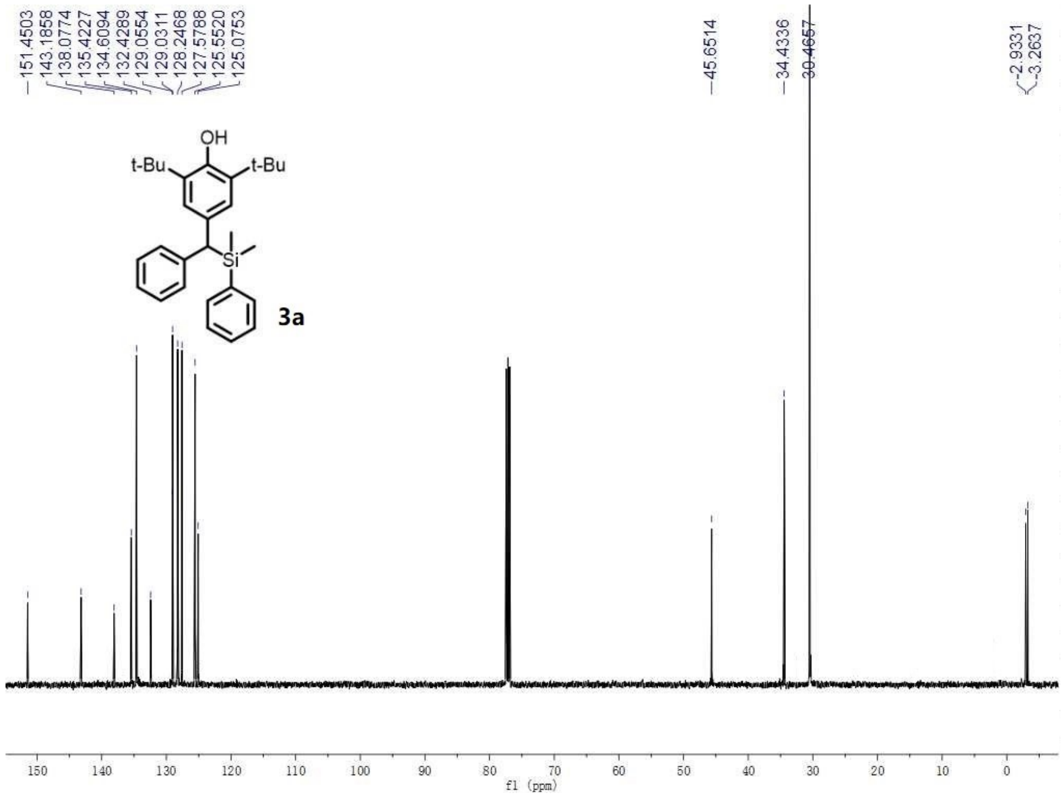

^{1}H NMR Spectrum of compound 3b

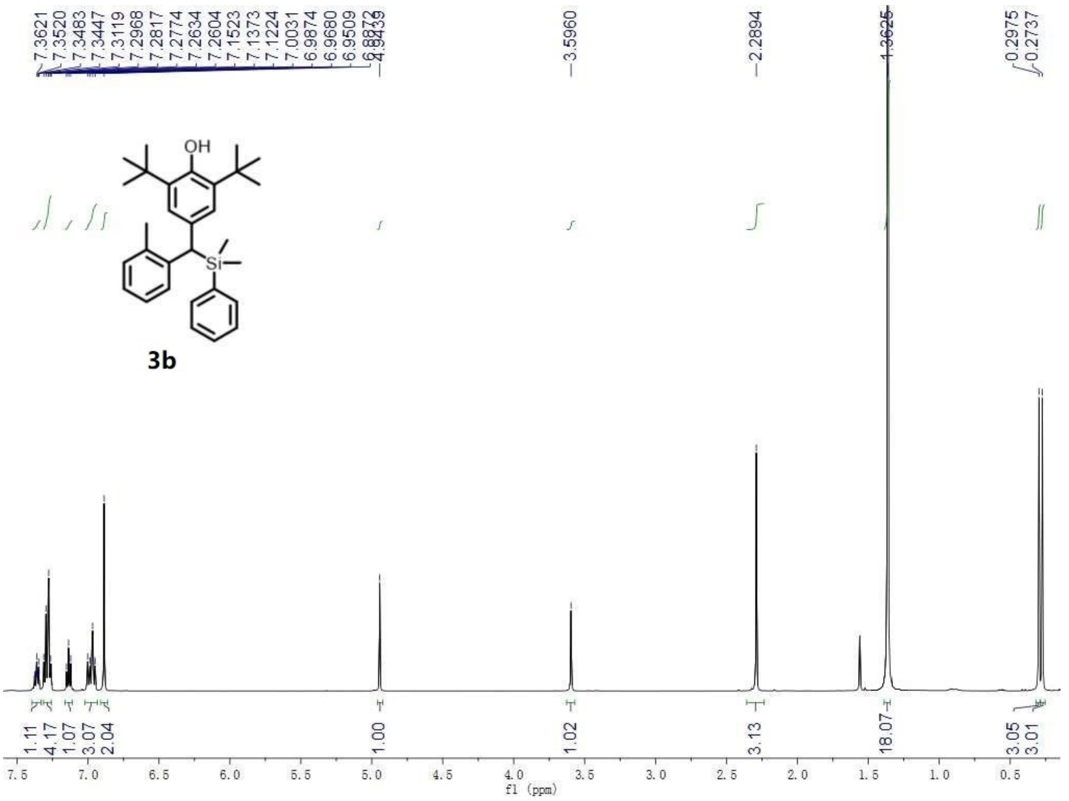

^{13}C NMR Spectrum of compound 3b

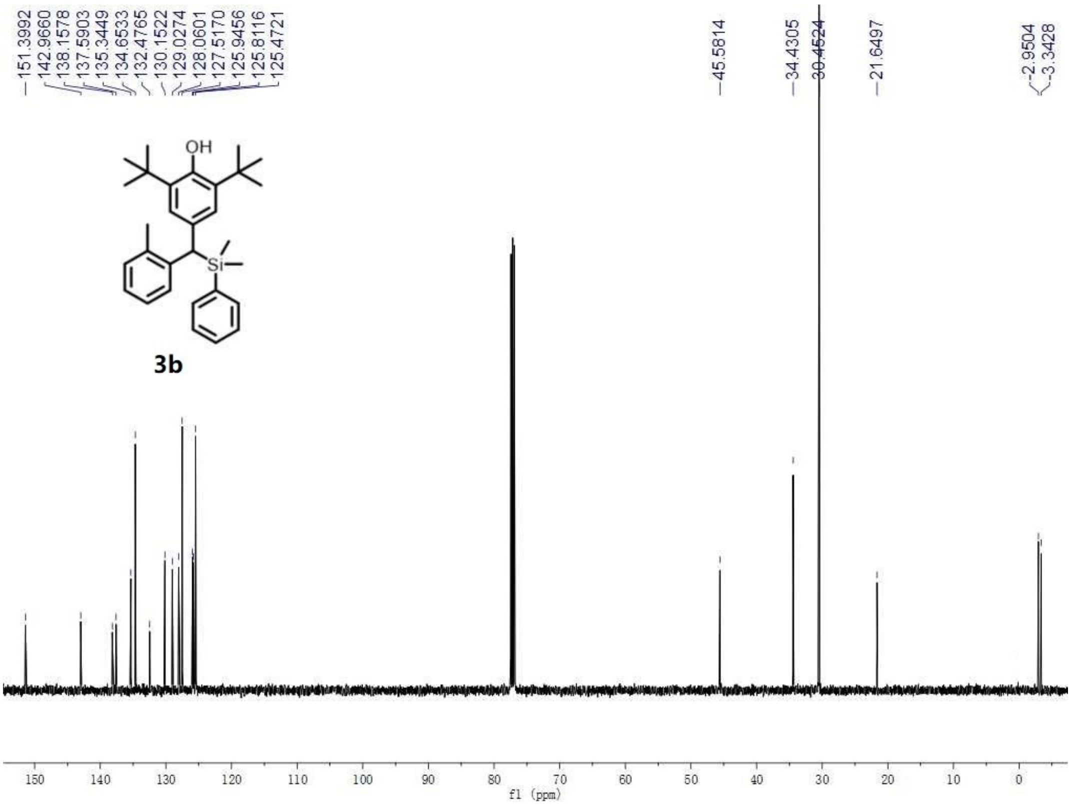

^{1}H NMR Spectrum of compound 3c

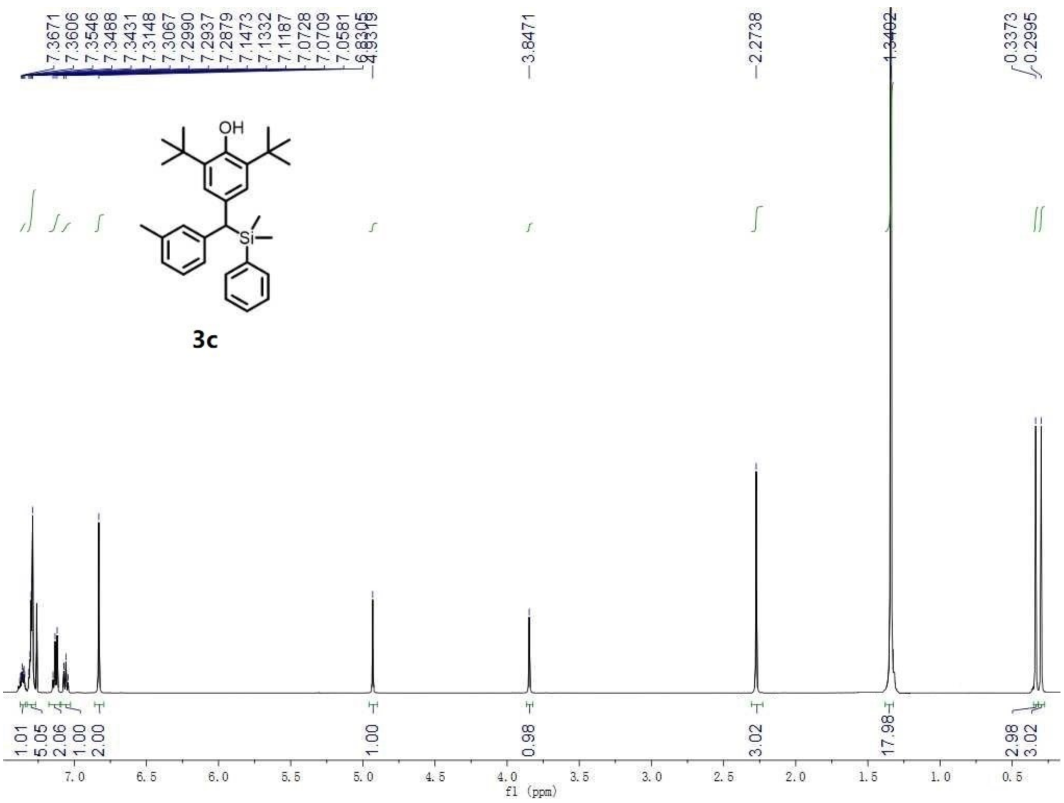

^{13}C NMR Spectrum of compound 3c

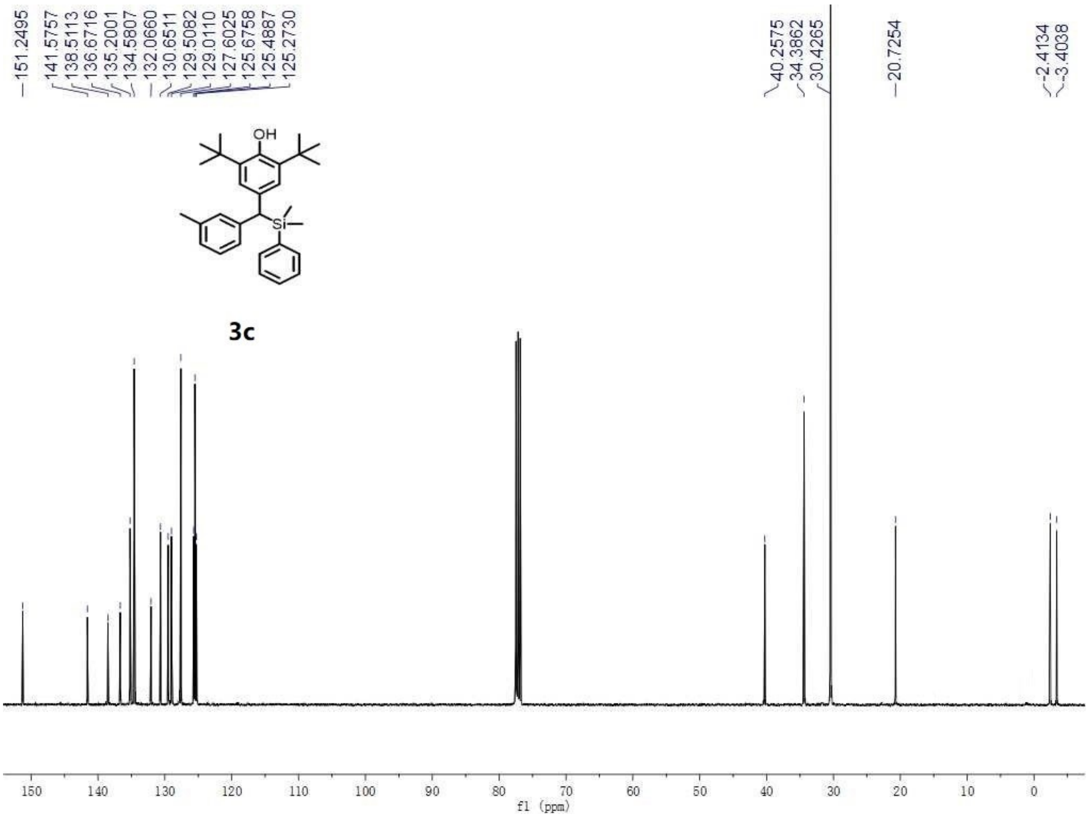

^{1}H NMR Spectrum of compound 3d

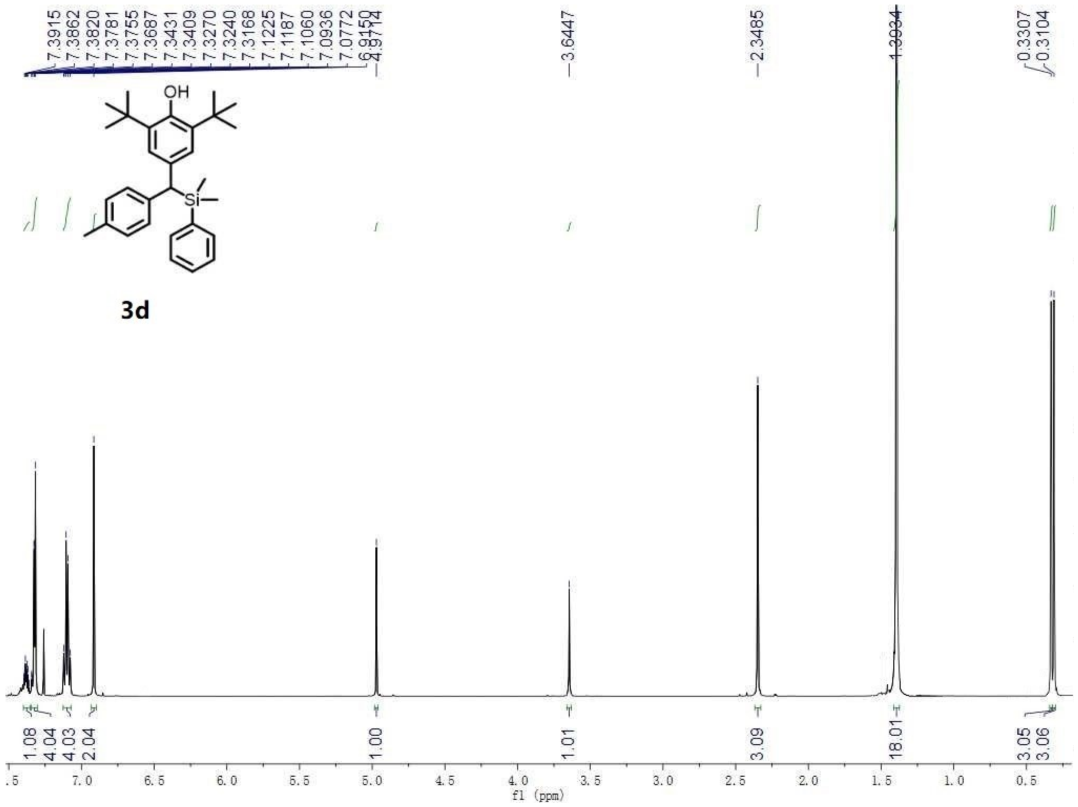

^{13}C NMR Spectrum of compound 3d

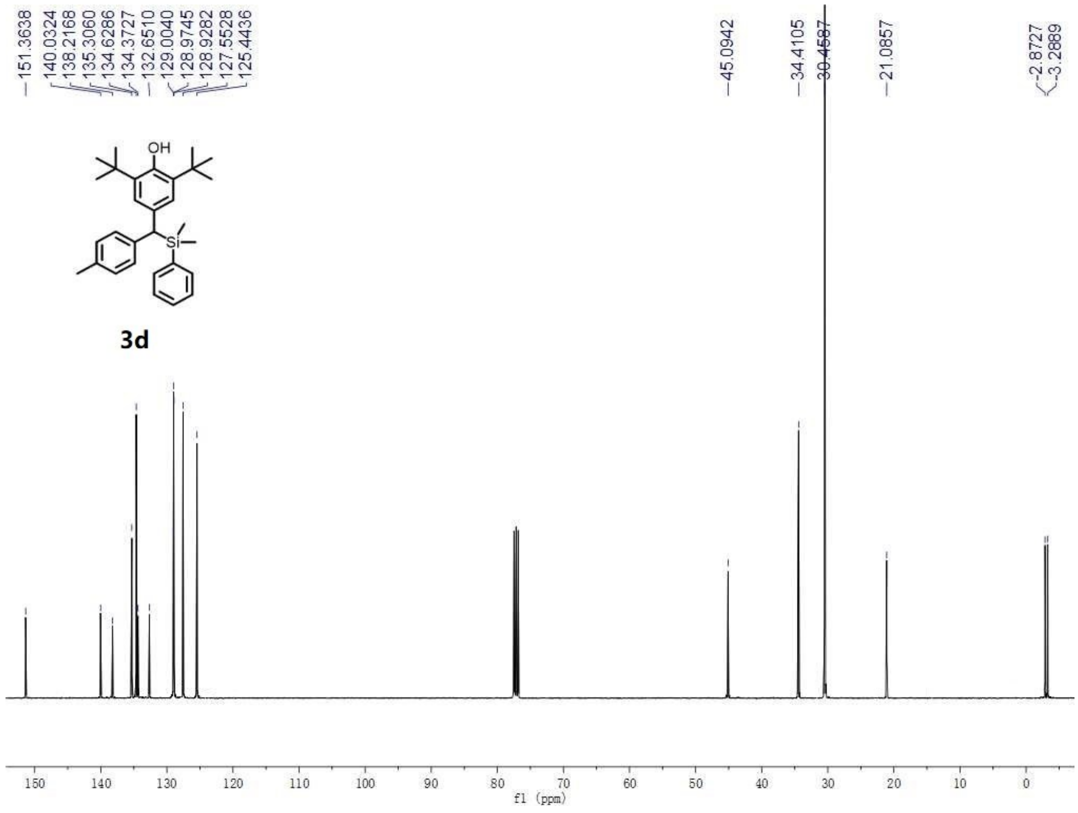

^{1}H NMR Spectrum of compound 3e

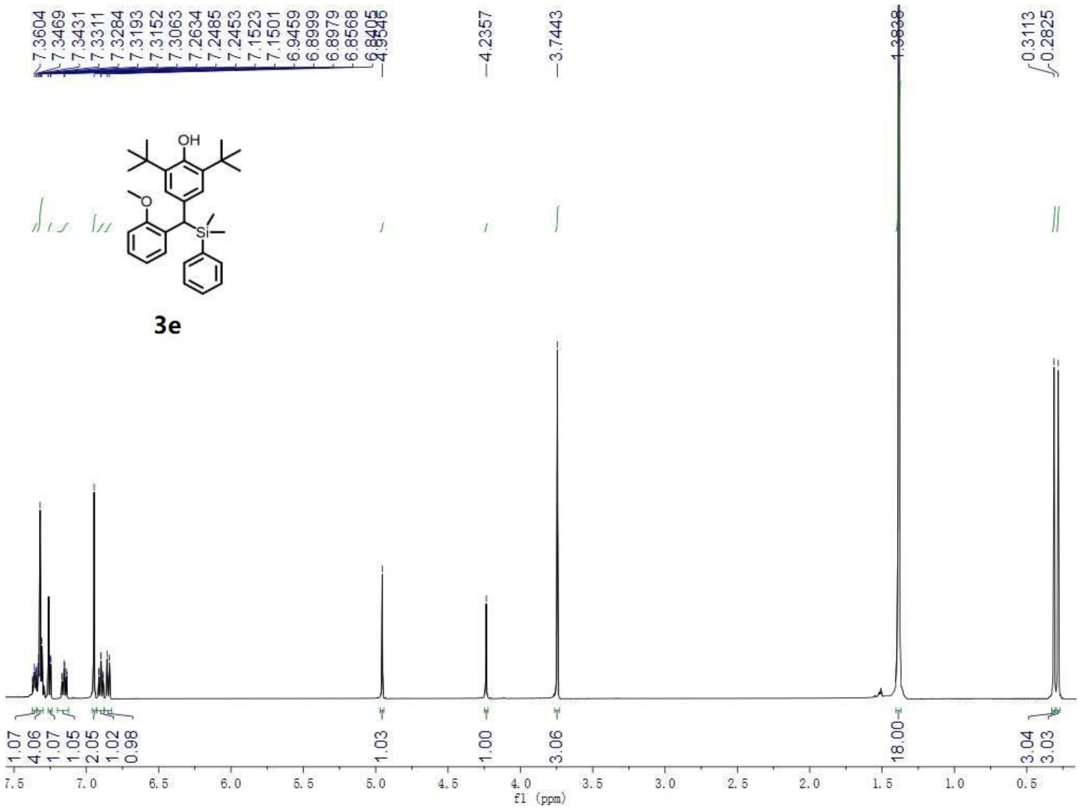

^{13}C NMR Spectrum of compound 3e

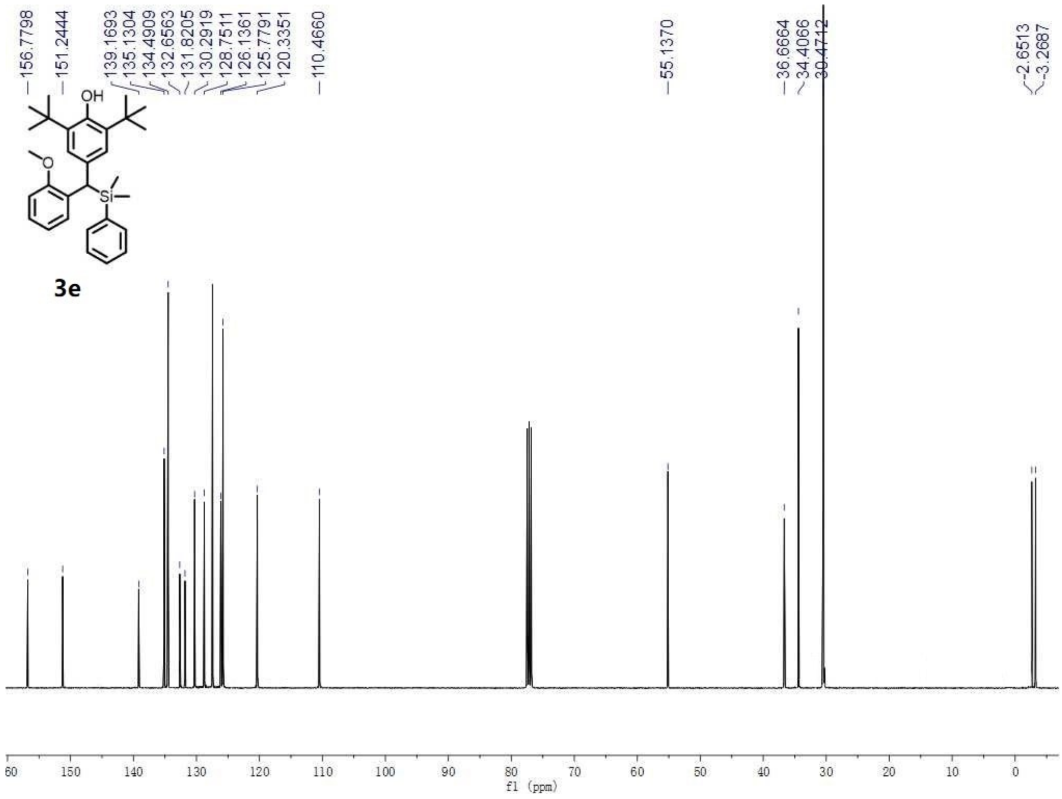

^{1}H NMR Spectrum of compound 3f

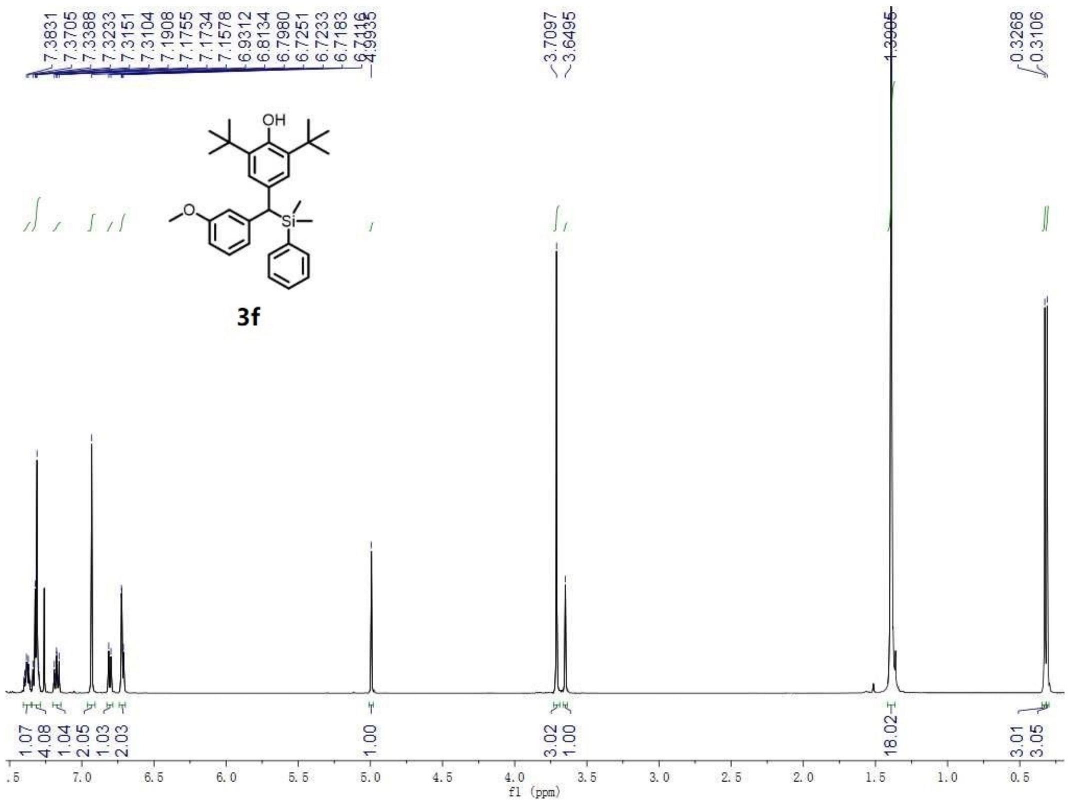

^{13}C NMR Spectrum of compound 3f

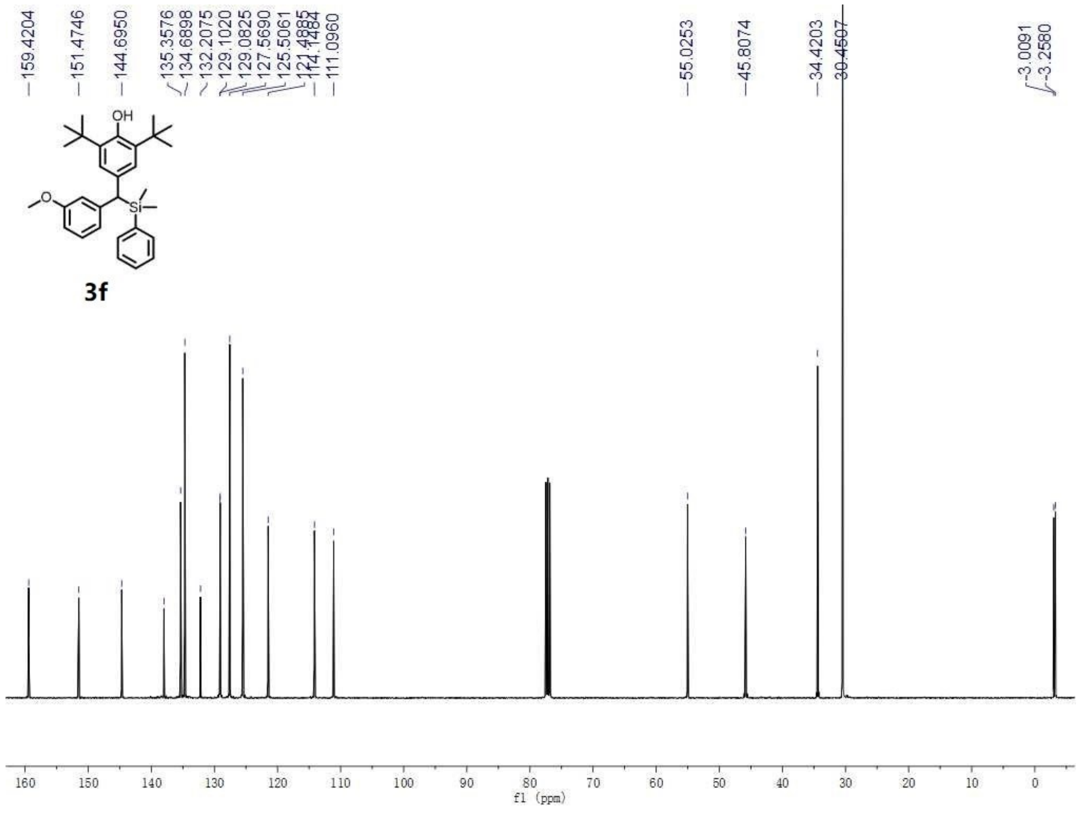

^{1}H NMR Spectrum of compound 3g

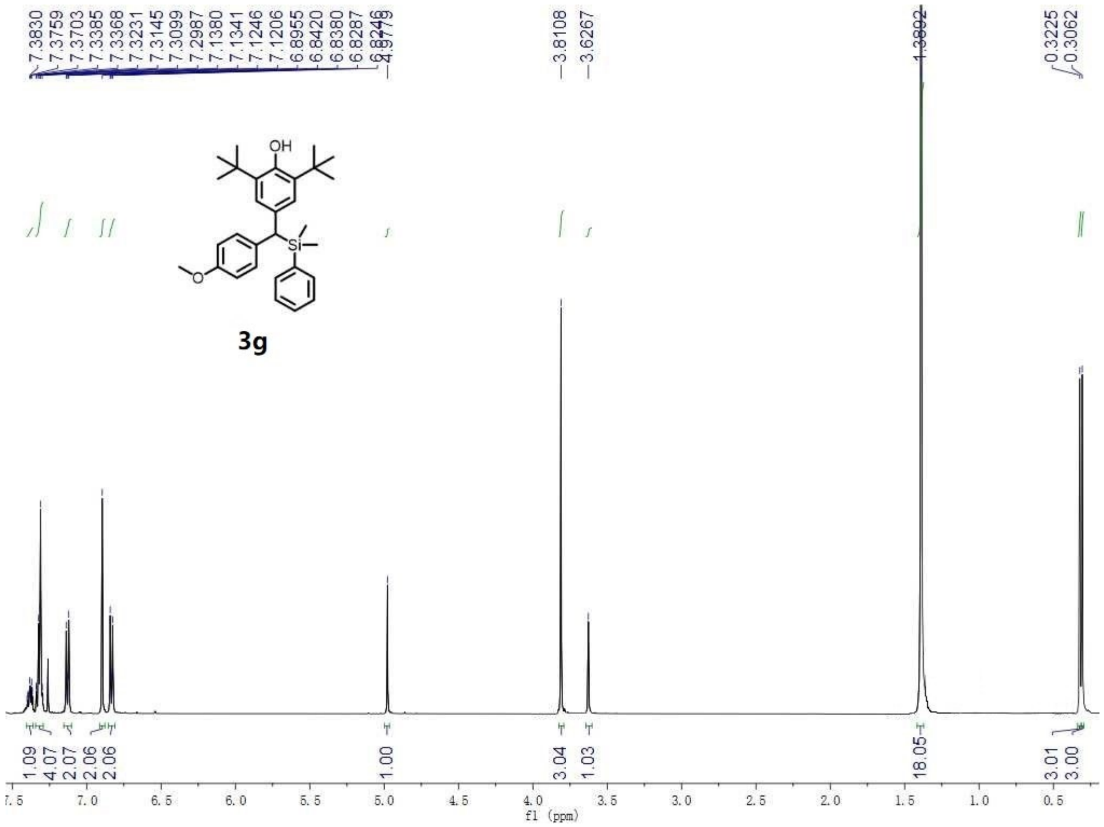

^{13}C NMR Spectrum of compound 3g

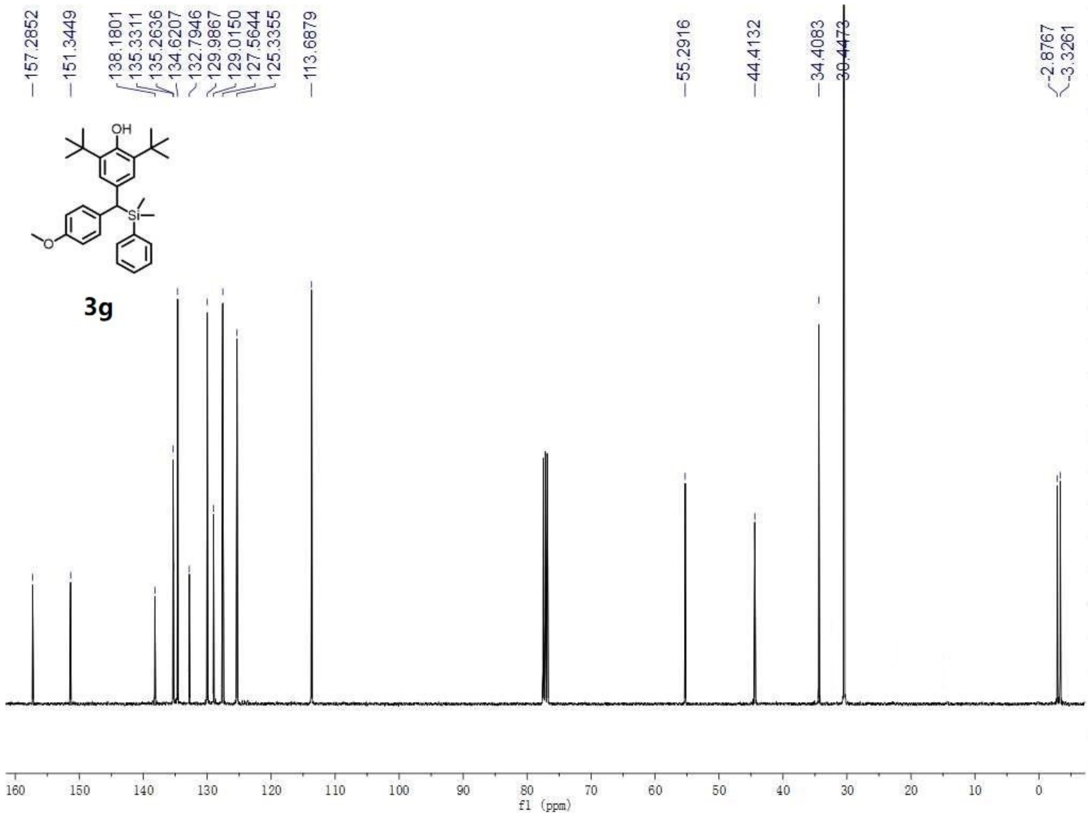

^{1}H NMR Spectrum of compound 3h

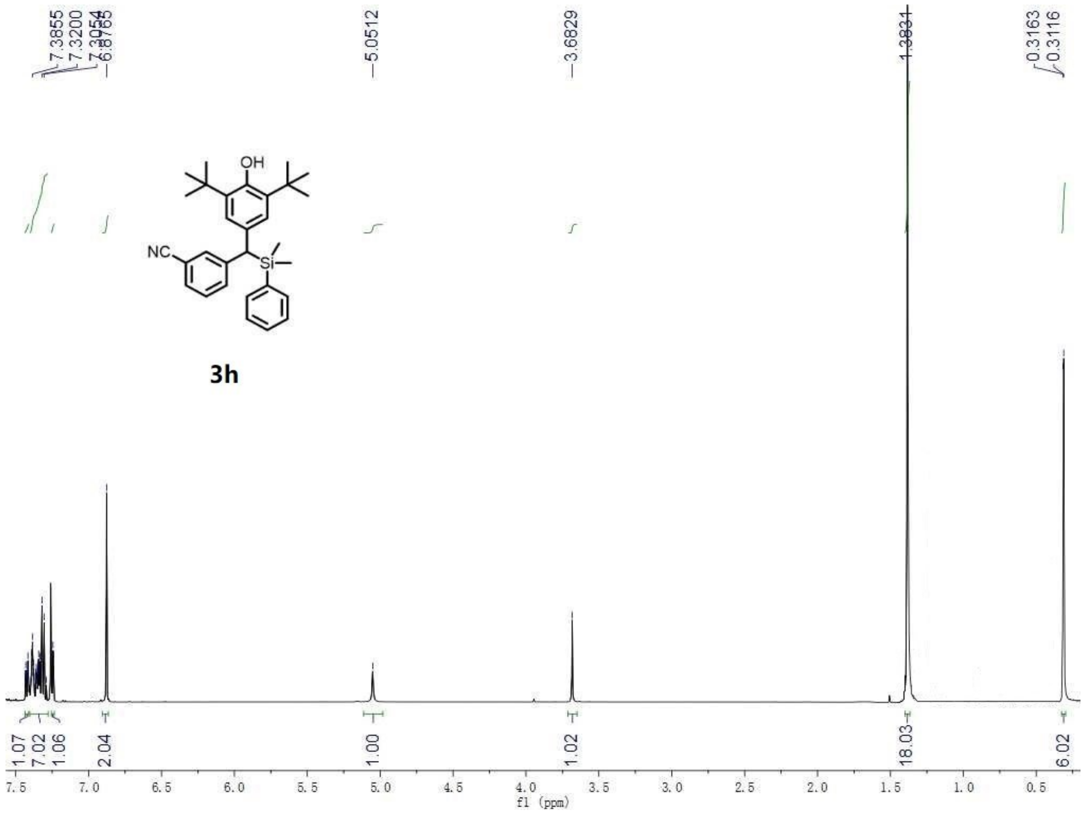

^{13}C NMR Spectrum of compound 3h

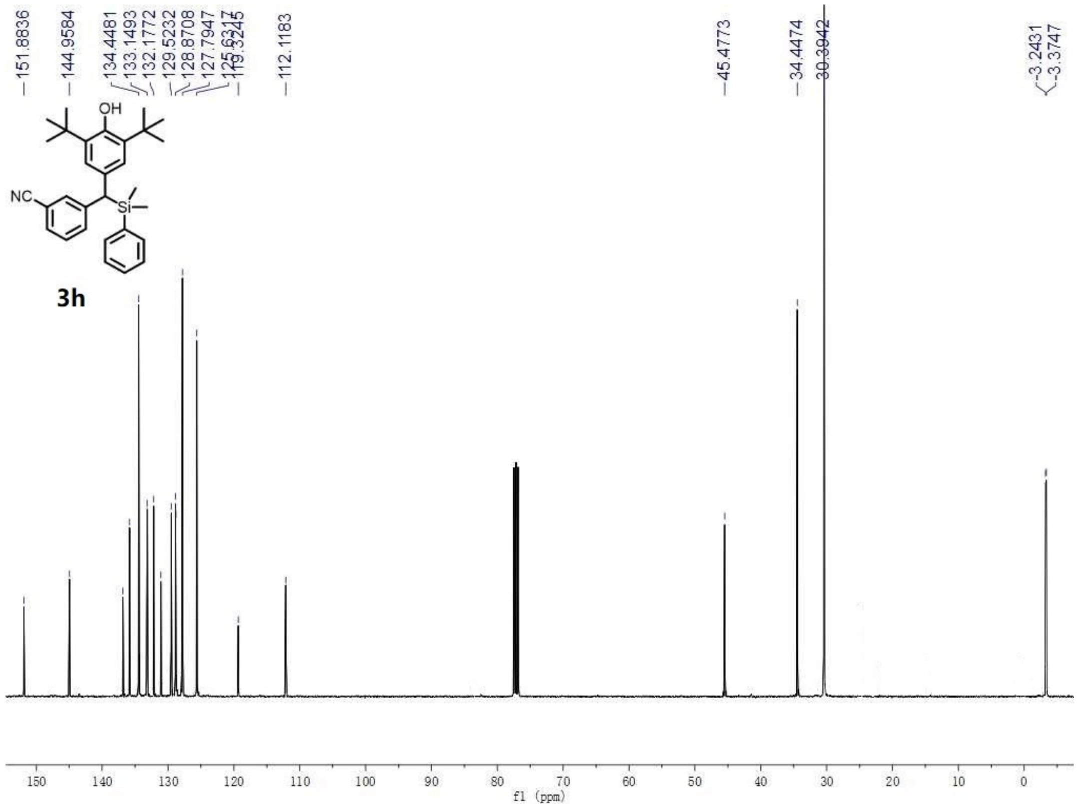

^{1}H NMR Spectrum of compound 3i

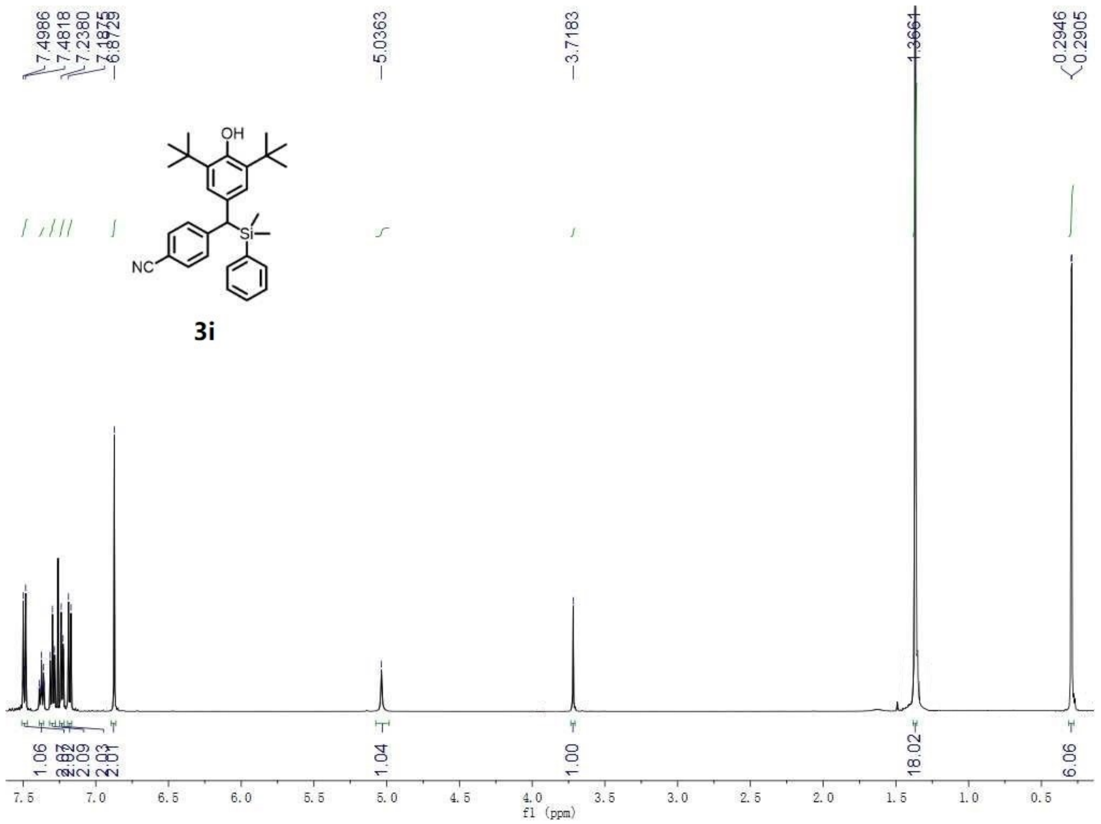

^{13}C NMR Spectrum of compound 3i

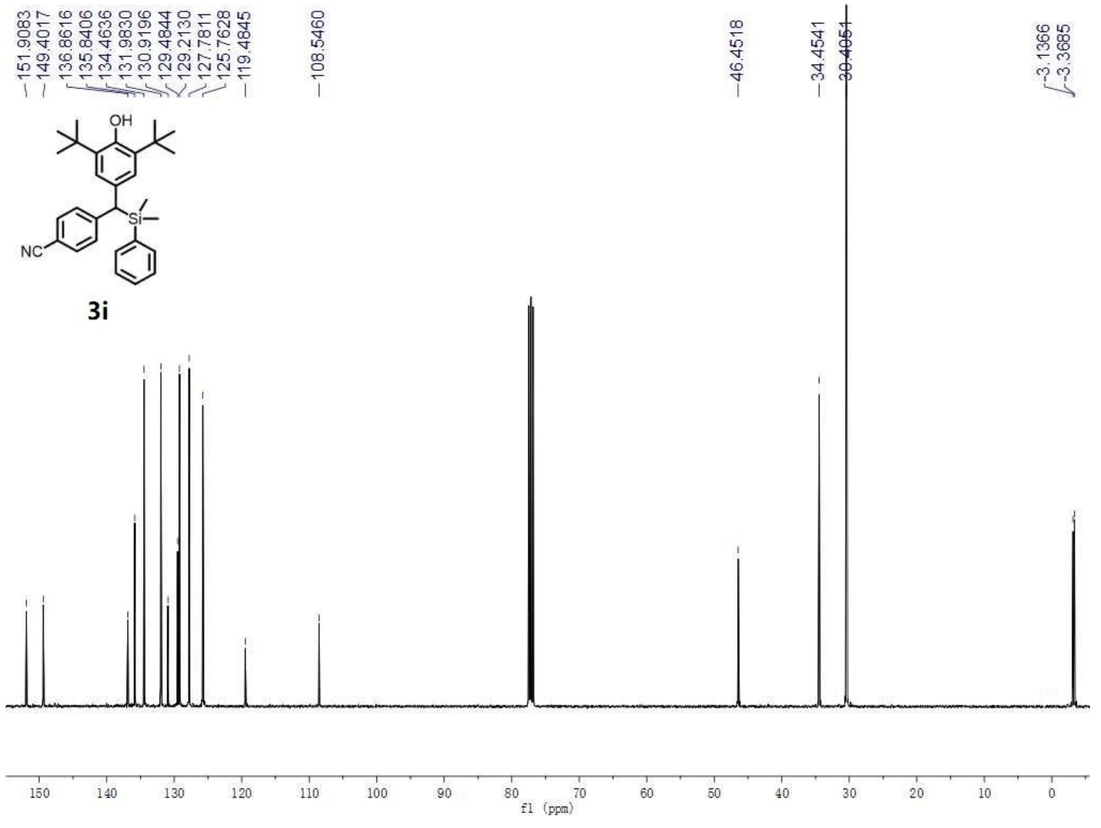

^{1}H NMR Spectrum of compound 3j

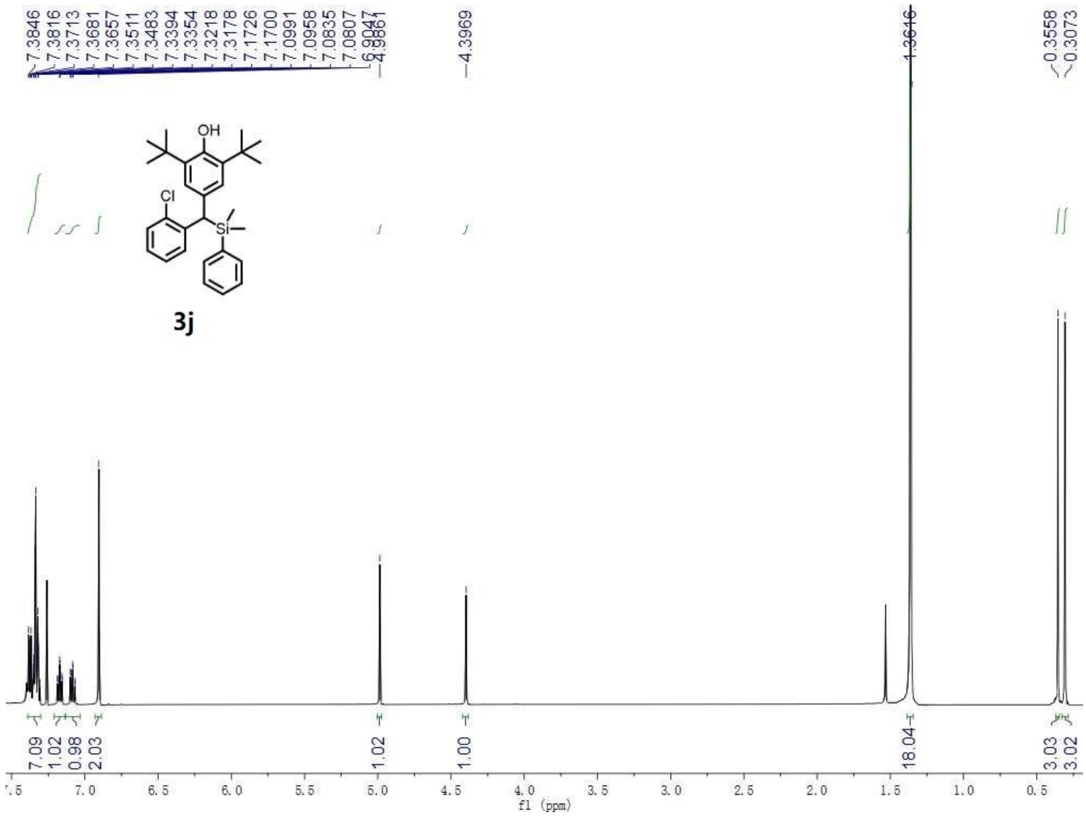

^{13}C NMR Spectrum of compound 3j

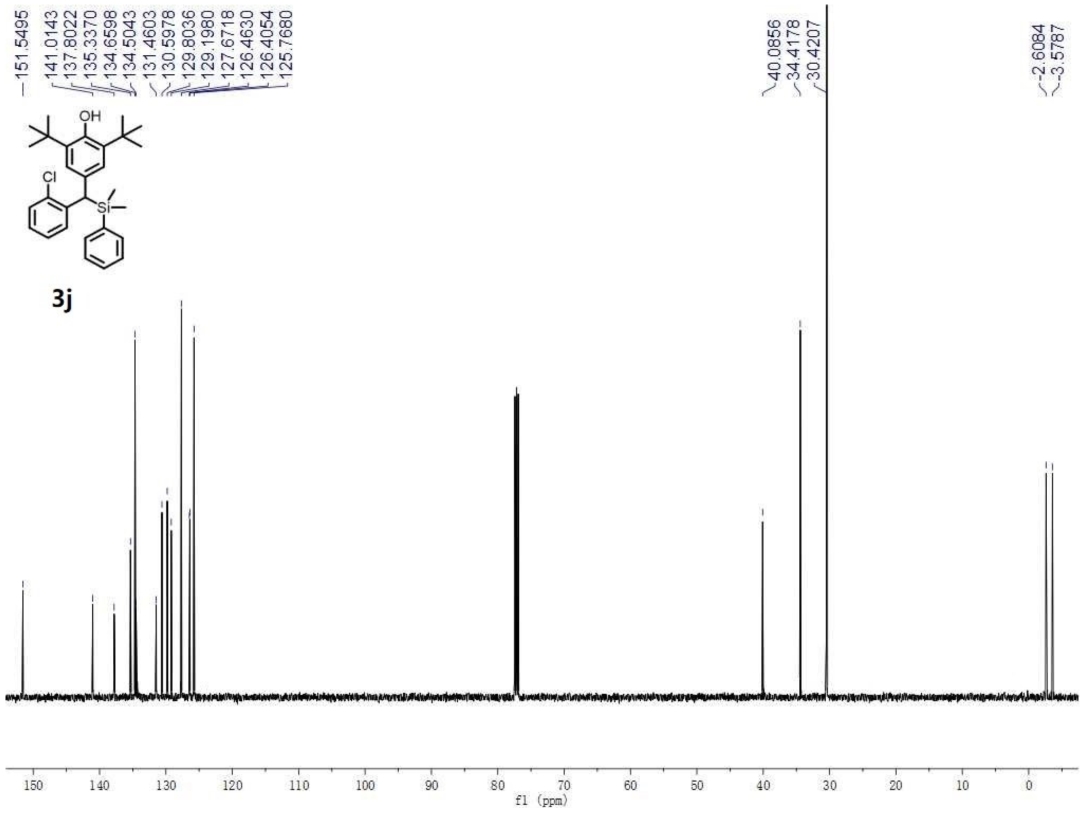

^{1}H NMR Spectrum of compound 3k

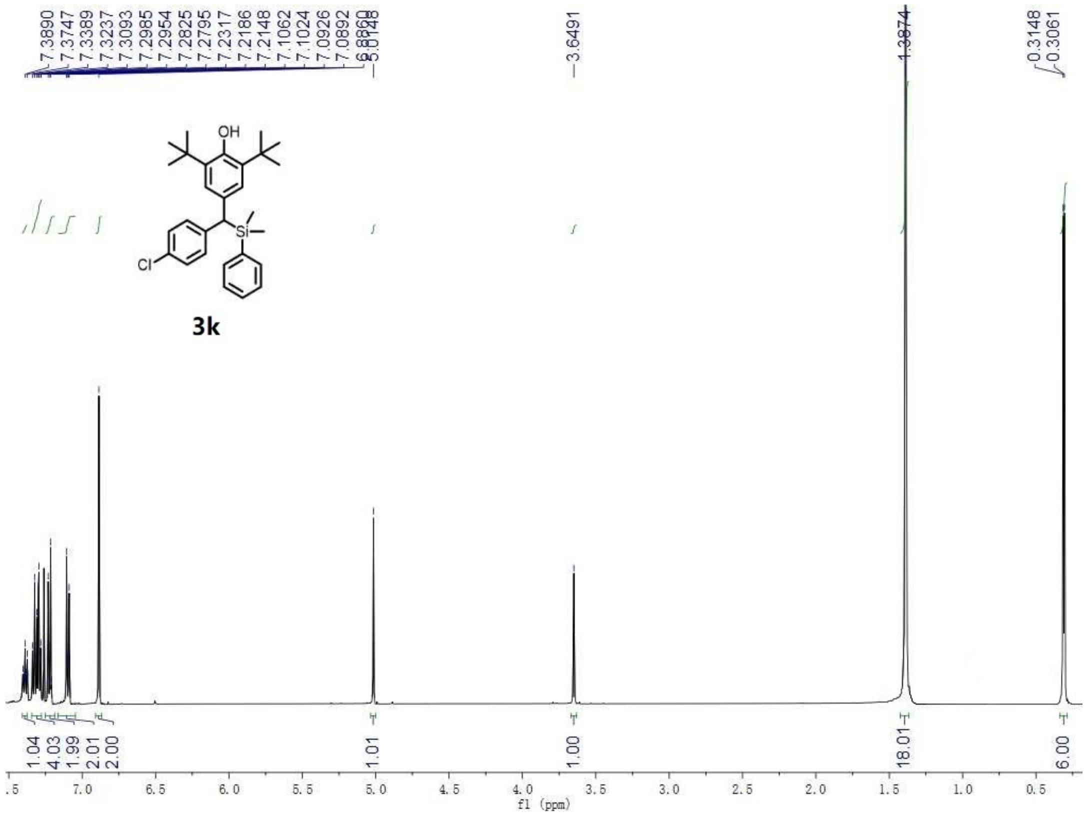

^{13}C NMR Spectrum of compound 3k

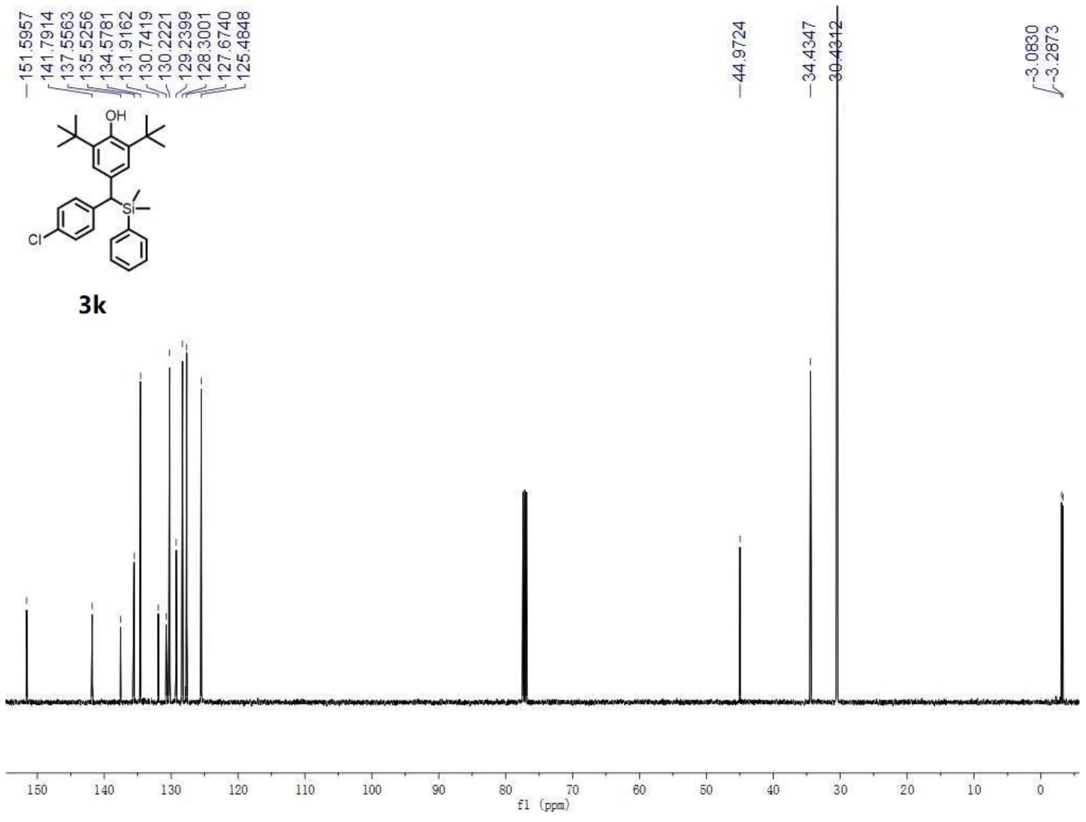

^1H NMR Spectrum of compound 3l

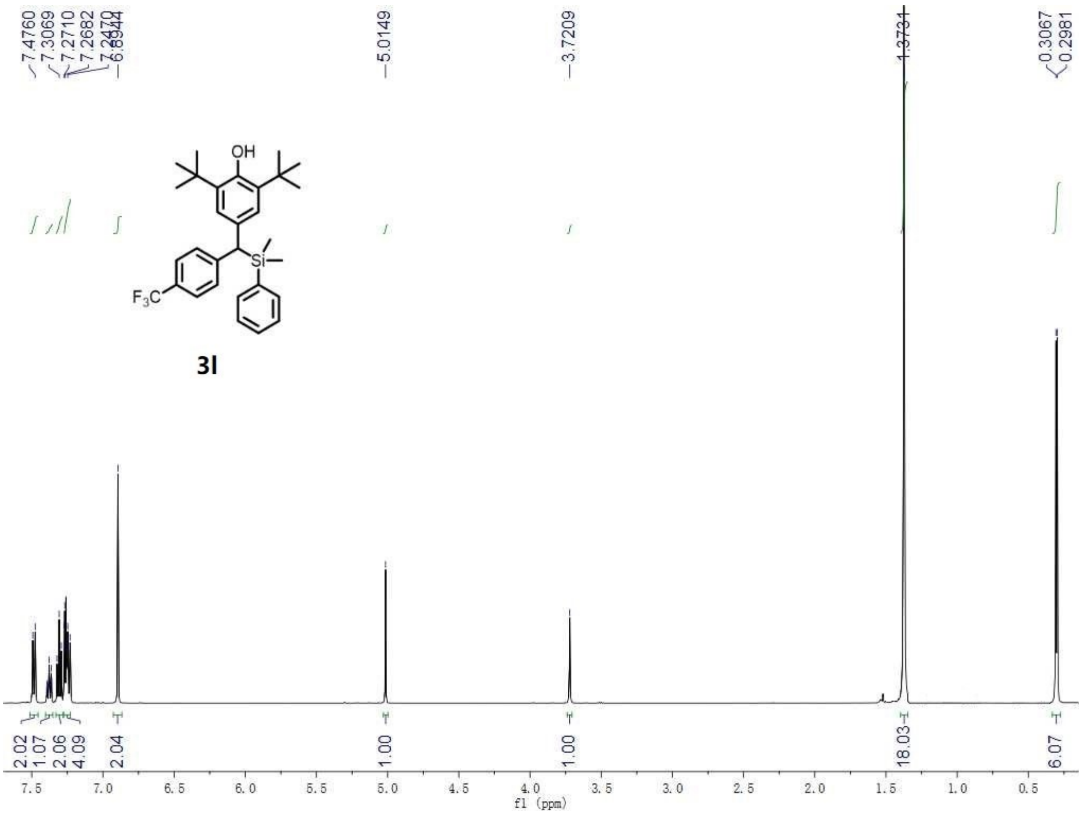

^{13}C NMR Spectrum of compound 3l

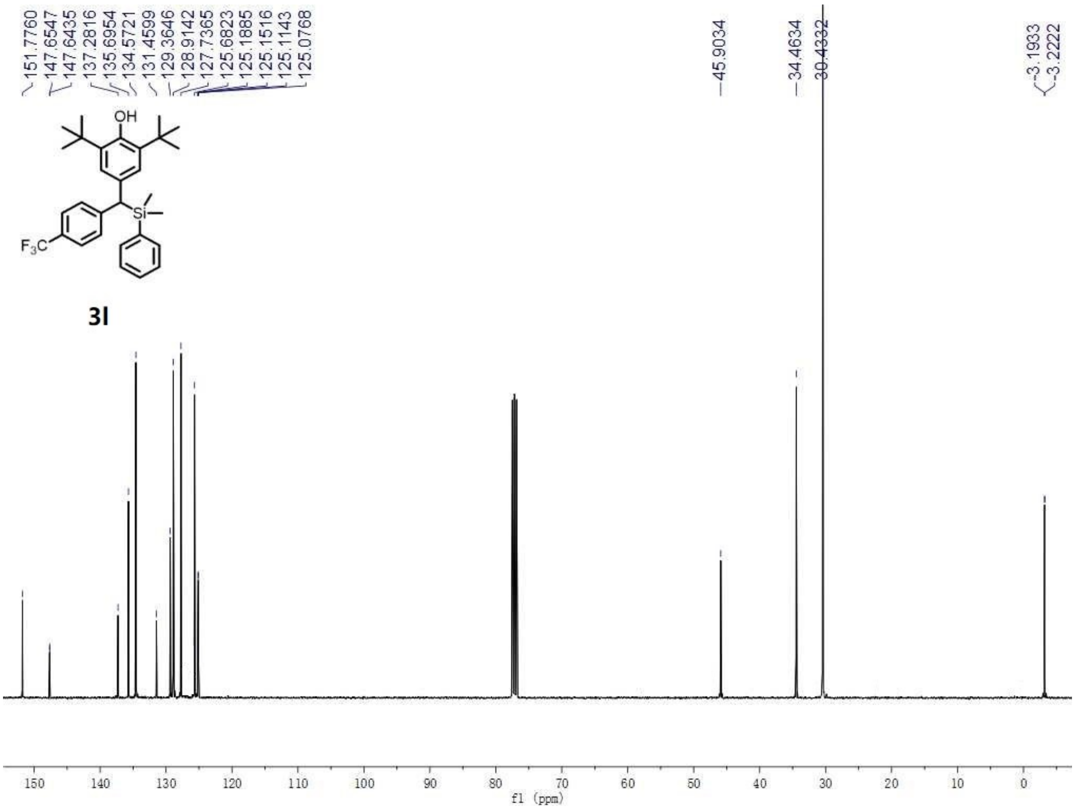

^{1}H NMR Spectrum of compound 3m

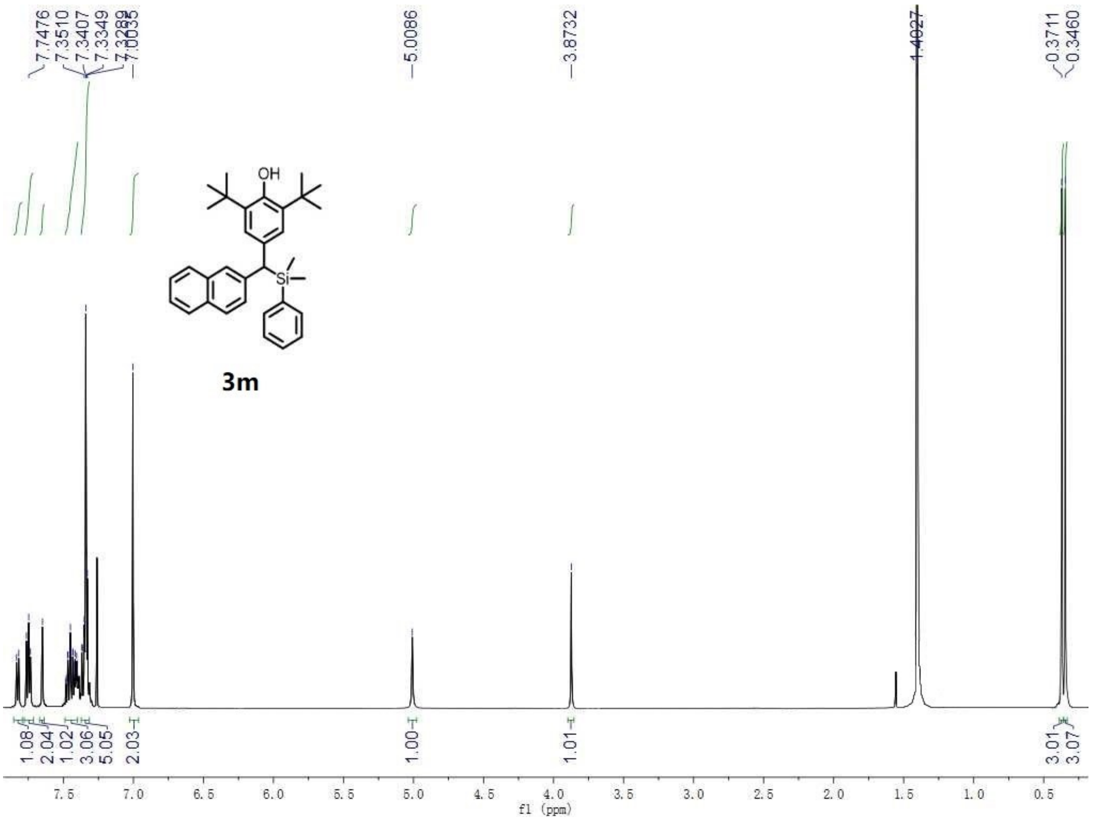

^{13}C NMR Spectrum of compound 3m

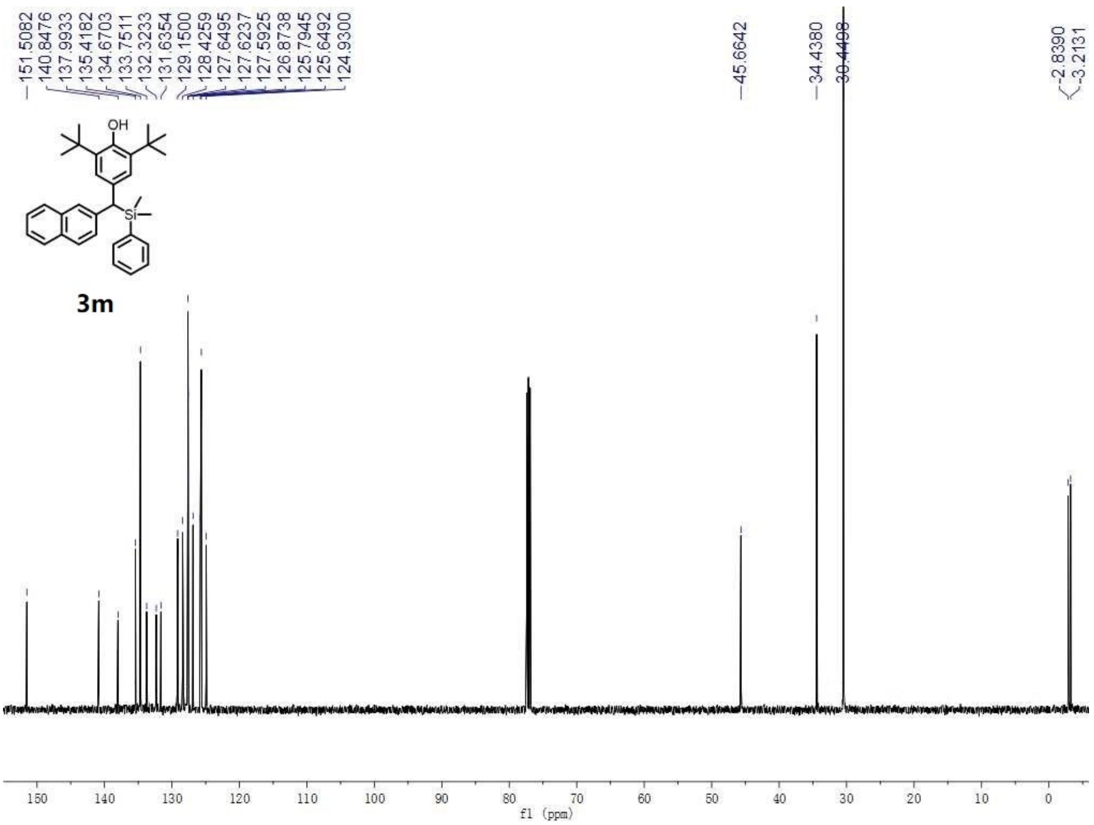

¹H NMR Spectrum of compound 3n

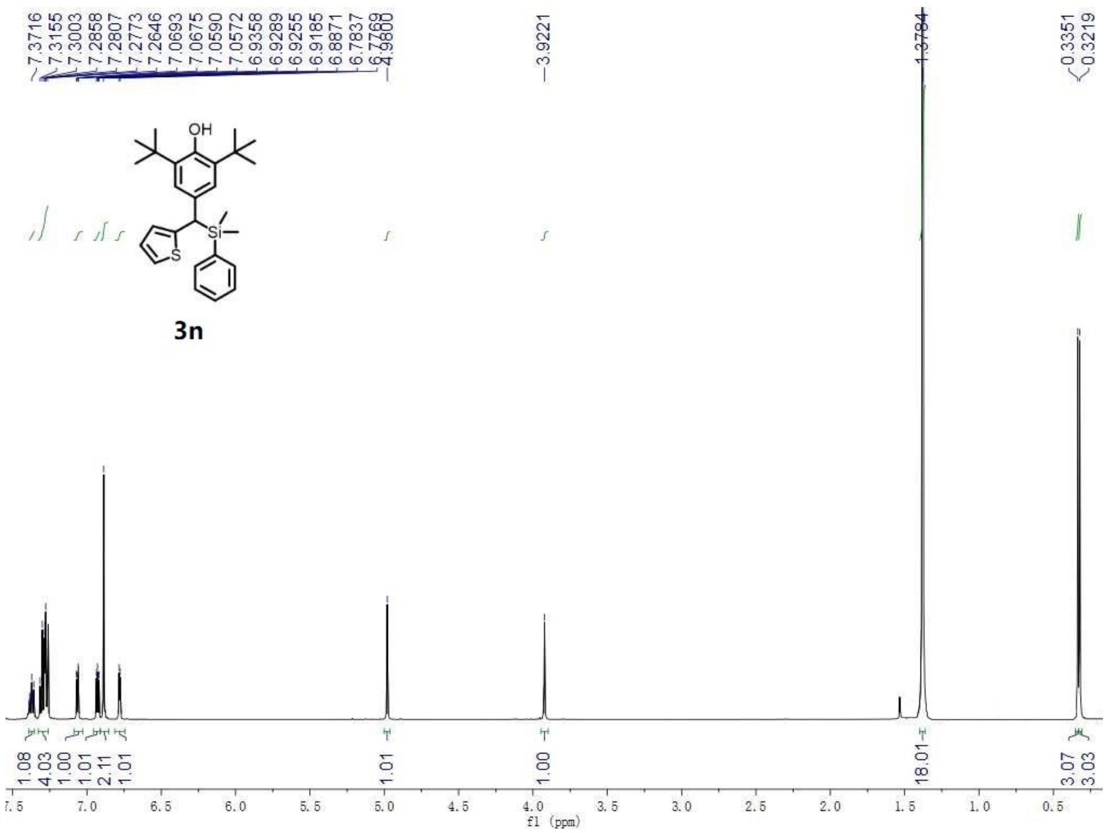

¹³C NMR Spectrum of compound 3n

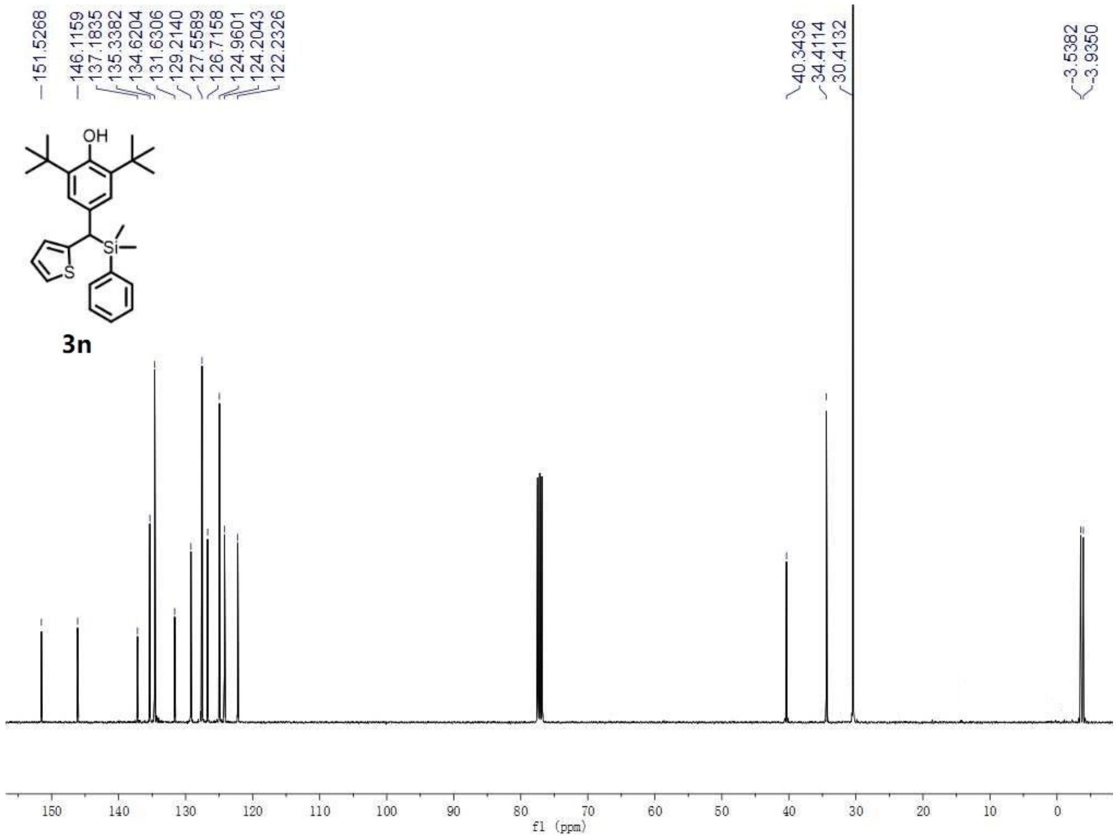

^{1}H NMR Spectrum of compound 3o

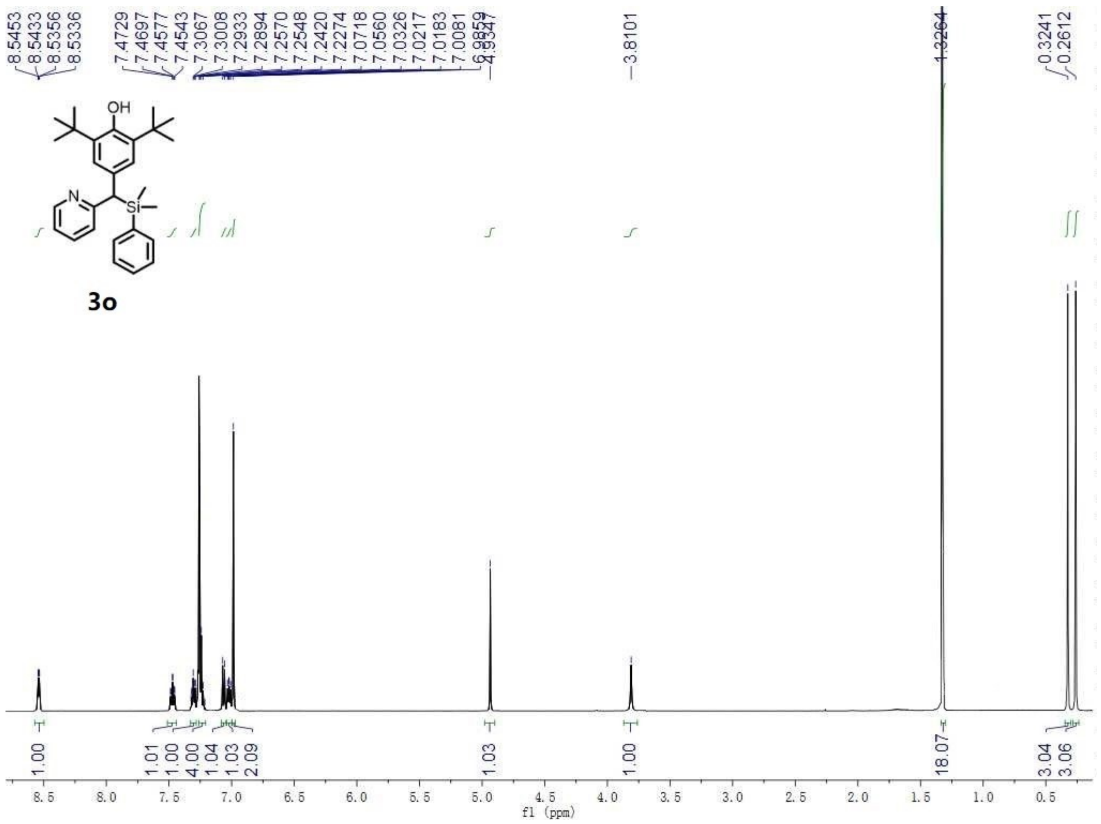

^{13}C NMR Spectrum of compound 3o

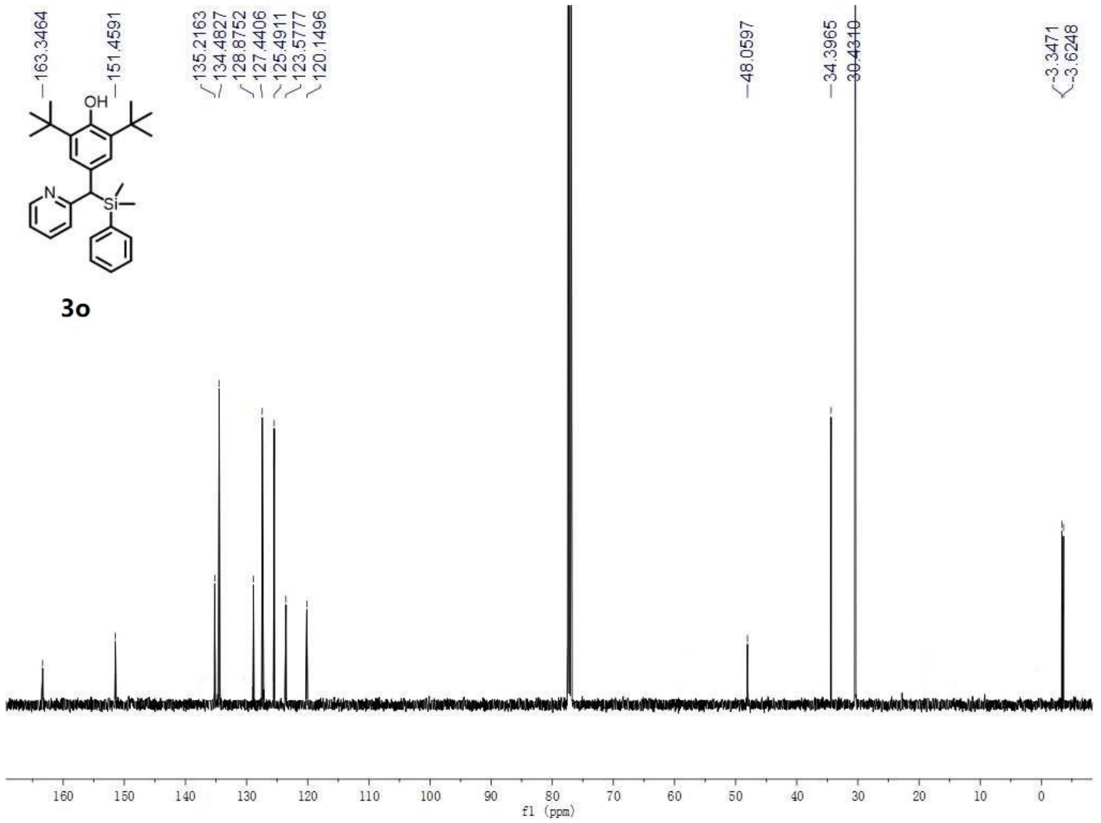

^{1}H NMR Spectrum of compound 3p

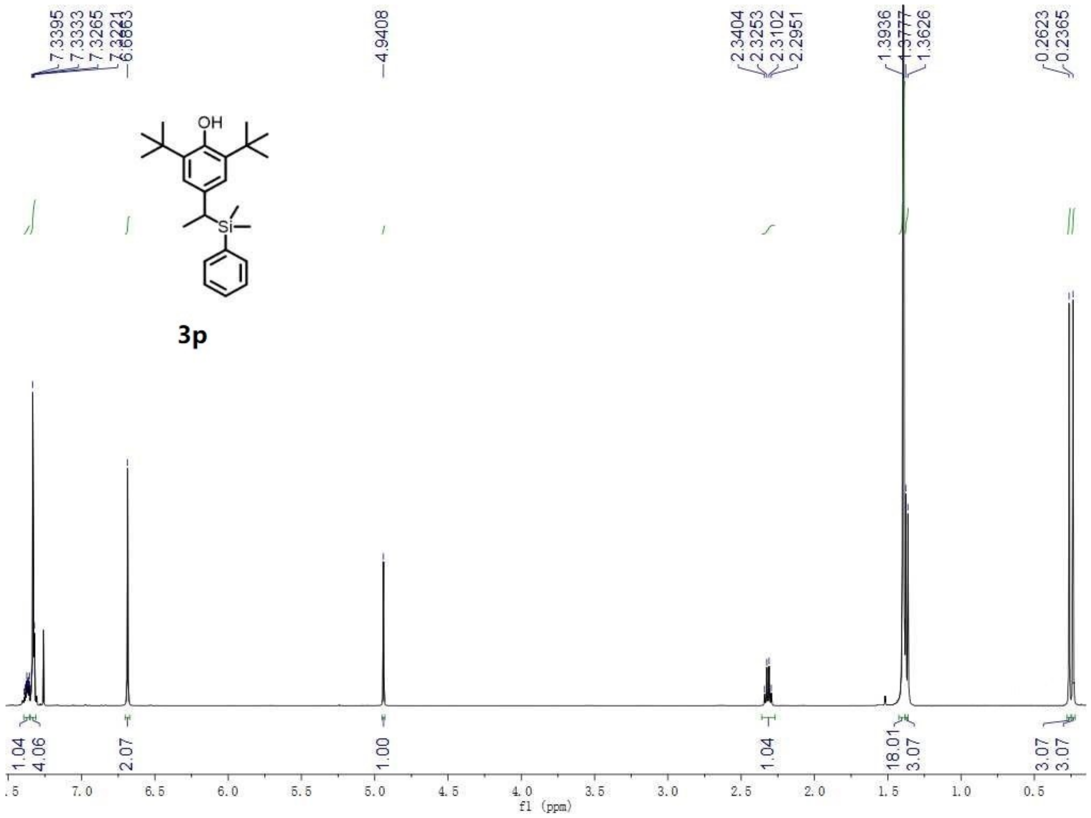

^{13}C NMR Spectrum of compound 3p

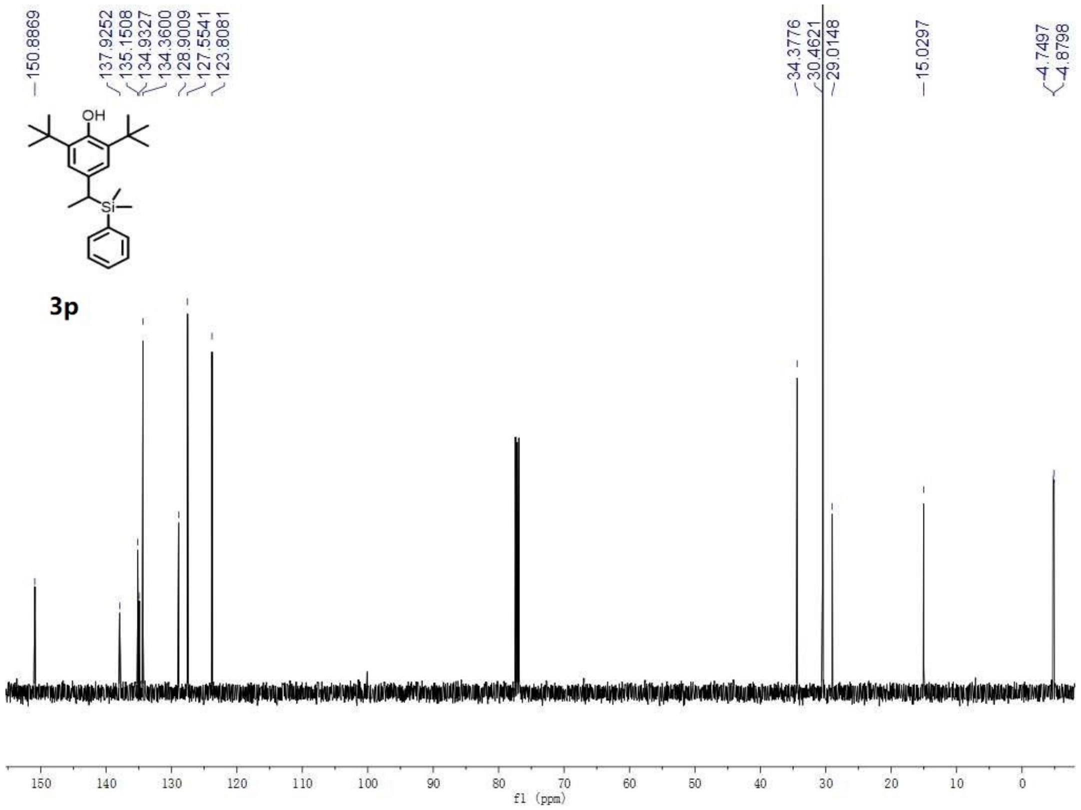

^{1}H NMR Spectrum of compound 4

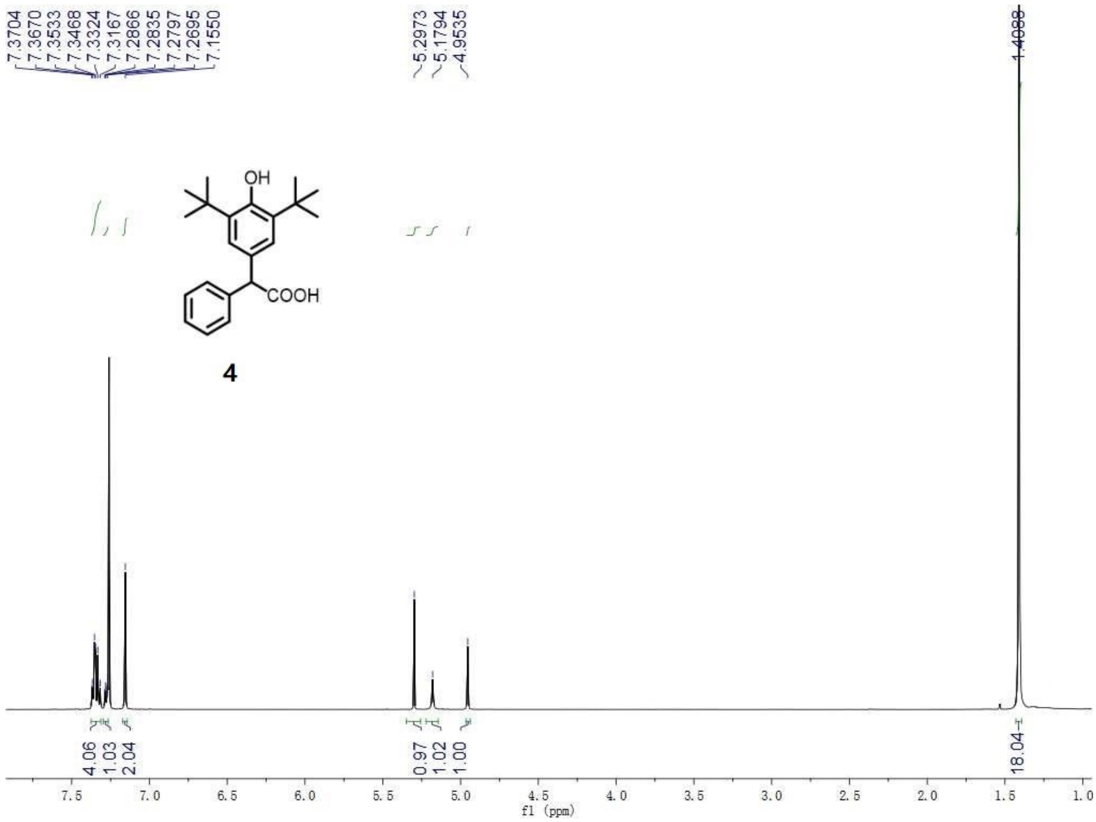

^{13}C NMR Spectrum of compound 4

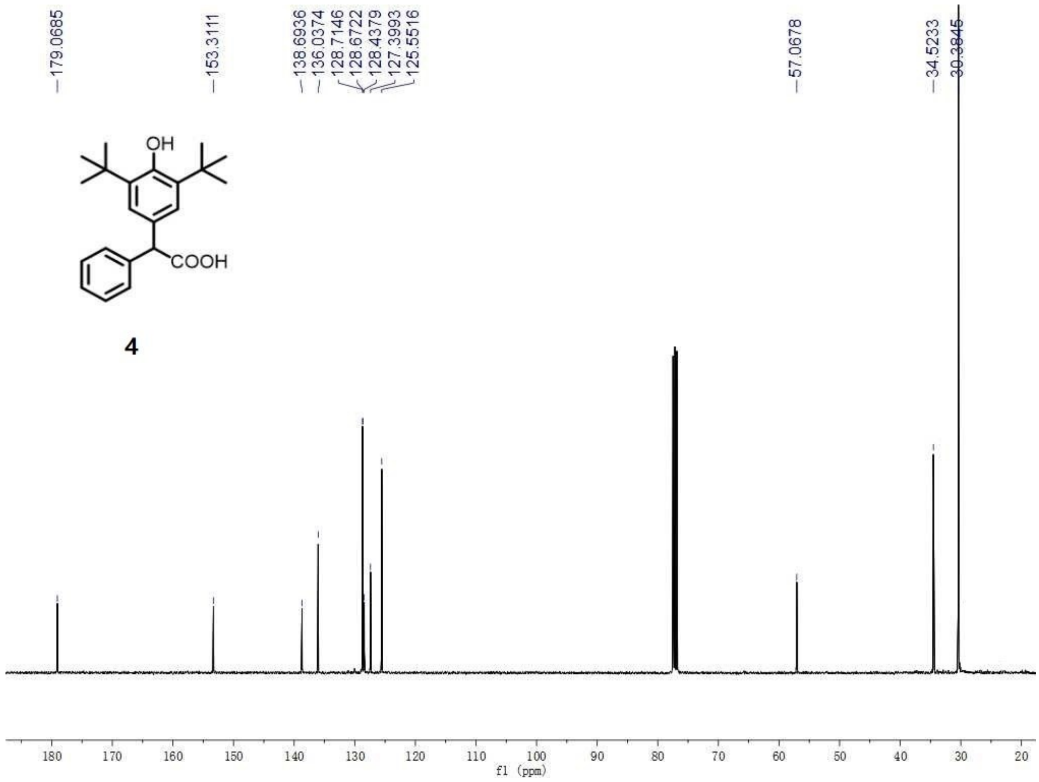

Supplement: RA-011-D1RA03193G-s001 [file RA-011-D1RA03193G-s001.pdf]
